# Economic and cost-effectiveness analysis of the Community-Level Interventions for Pre-eclampsia (CLIP) trials in India, Pakistan and Mozambique

**DOI:** 10.1136/bmjgh-2020-004123

**Published:** 2021-05-24

**Authors:** Jeffrey N Bone, Asif R Khowaja, Marianne Vidler, Beth A Payne, Mrutyunjaya B Bellad, Shivaprasad S Goudar, Ashalata A Mallapur, Khatia Munguambe, Rahat N Qureshi, Charfudin Sacoor, Esperanca Sevene, Geert W J Frederix, Zulfiqar A Bhutta, Craig Mitton, Laura A Magee, Peter von Dadelszen, the CLIP Trials Working Group

**Affiliations:** 1 Department of Obstetrics and Gynaecology, The University of British Columbia, Vancouver, British Columbia, Canada; 2 School of Population and Public Health, The University of British Columbia, Vancouver, British Columbia, Canada; 3 Women’s and Children’s Health Research Unit, KLE Academy of Higher Education and Research, Belgaum, Karnataka, India; 4 S Nijalingappa Medical College and HSK Hospital and Research Centre, Bagalkot, Karnataka, India; 5 Centro de Investigação em Saúde de Manhiça, Manhiça, Maputo, Mozambique; 6 Centre of Excellence, Division of Woman and Child Health, Aga Khan University, Karachi, Pakistan; 7 Universidade Eduardo Mondlane, Maputo, Mozambique; 8 Julius Center for Health Sciences and Primary Care, Utrecht, The Netherlands; 9 Centre for Global Child Health, Hospital for Sick Children, Toronto, Ontario, Canada; 10 Department of Women and Children's Health, School of Life Course Sciences, King's College London, London, UK

**Keywords:** health economics, maternal health, obstetrics, hypertension, intervention study

## Abstract

**Background:**

The Community-Level Interventions for Pre-eclampsia (CLIP) trials (NCT01911494) in India, Pakistan and Mozambique (February 2014–2017) involved community engagement and task sharing with community health workers for triage and initial treatment of pregnancy hypertension. Maternal and perinatal mortality was less frequent among women who received ≥8 CLIP contacts. The aim of this analysis was to assess the incremental costs and cost-effectiveness of the CLIP intervention overall in comparison to standard of care, and by PIERS (Pre-eclampsia Integrated Estimate of RiSk) On the Move (POM) mobile health application visit frequency.

**Methods:**

Included were all women enrolled in the three CLIP trials who had delivered with known outcomes by trial end. According to the number of POM-guided home contacts received (0, 1–3, 4–7, ≥8), costs were collected from annual budgets and spending receipts, with inclusion of family opportunity costs in Pakistan. A decision tree model was built to determine the cost-effectiveness of the intervention (vs usual care), based on the primary clinical endpoint of years of life lost (YLL) for mothers and infants. A probabilistic sensitivity analysis was used to assess uncertainty in the cost and clinical outcomes.

**Results:**

The incremental per pregnancy cost of the intervention was US$12.66 (India), US$11.51 (Pakistan) and US$13.26 (Mozambique). As implemented, the intervention was not cost-effective due largely to minimal differences in YLL between arms. However, among women who received ≥8 CLIP contacts (four in Pakistan), the probability of health system and family (Pakistan) cost-effectiveness was ≥80% (all countries).

**Conclusion:**

The intervention was likely to be cost-effective for women receiving ≥8 contacts in Mozambique and India, and ≥4 in Pakistan, supporting WHO guidance on antenatal contact frequency.

**Trial registration number:**

NCT01911494.

**Key questions**

**What is already known?**

- Recent reviews have indicated that many community health worker-led initiatives are cost-effective for maternal, neonatal and child health outcomes in low and middle-income countries (LMICs).
- Health economic analyses relating to interventions for the hypertensive disorders of pregnancy have been based primarily on diagnostic and clinical interventions.

**What are the new findings?**

- The Community-Level Interventions for Pre-eclampsia (CLIP) trials are the first to undertake a solely community-level intervention for pregnancy hypertension.
- Consistent with the primary trial data which demonstrated no overall benefit, the CLIP intervention was not cost-effective as implemented.
- When women received at least eight home-based visits from community health workers, there was a cost-effective reduction in a composite of maternal and perinatal mortality and morbidity, driven primarily by perinatal mortality.
- Data were broadly consistent across three diverse LMIC settings (in India, Pakistan and Mozambique), and provide support for the cost-effectiveness of community health worker-led interventions when staffing levels can support sufficient coverage of the population and frequency of contacts.

**What do the new findings imply?**

- The CLIP trials were not a cost-effective approach to reducing adverse maternal and perinatal outcomes as implemented, but our findings of increasing cost-effectiveness with higher frequency of antenatal contacts support community-level intervention if scalability can be achieved.
- Our data support a role for community health workers in delivering the eight antenatal care contacts model advocated by the WHO.

## Introduction

The hypertensive disorders of pregnancy are one of the primary global causes of maternal and fetal mortality^1^ and morbidity, with pre-eclampsia specifically resulting in an estimated 76 000 maternal and 500 000 perinatal deaths annually.^2^ Recently, using data from the Community-Level Interventions for Pre-eclampsia (CLIP) cluster randomised controlled trials (cRCTs) in India, Pakistan, Mozambique and Nigeria, we estimated the incidence of pregnancy hypertension in low and middle-income countries (LMIC) to be at least 10%, at least as high as in well-resourced settings.^3^

Many of the pregnancy hypertension-related deaths occur either prior to women arriving at a health facility or after arriving there too late to prevent a fatal complication.^4^ As a result, there has been interest in mobilising front-line providers, such community healthcare workers (CHWs) to provide earlier care and facilitate referral to facility.^5 6^ However, it has not been demonstrated that such an approach was effective or cost-effective.

The CLIP cRCTs in India, Pakistan and Mozambique leveraged the existing CHW workforce to identify, implement initial treatment and triage hypertensive pregnant women in their communities to facility, as well as to provide education to communities about obstetric emergencies and the hypertensive disorders of pregnancy specifically. There was no evidence that the CLIP intervention was effective in decreasing a composite of maternal and perinatal mortality and morbidity as implemented by the existing workforce. Consequently, the intervention was received by fewer women than anticipated (ie, 7055 (90.0%) in India, 11 399 (56.3%) in Pakistan and 4809 (60.4%) in Mozambique).^7–10^ An a priori*-*determined contact frequency analysis indicated effectiveness in reducing maternal and perinatal mortality and morbidity among women who received the intervention per protocol; at least four visits in Pakistan, and at least eight visits in both India and Mozambique.^7–10^ These findings were consistent with the recent WHO eight antenatal care contacts model.

Health economic analyses in such global health trials are required, as they often provide policy direction for resource allocation for improving maternal and newborn health.^11^ Assessing cost-effectiveness of CHW-led interventions has been a matter of debate,^12^ and definitive determination of whether or not such an approach is a fiscally viable way of reducing adverse maternal and fetal outcomes is required.

The aim of this study was to assess the cost-effectiveness of the CLIP trial intervention in each of India, Pakistan and Mozambique in comparison to standard of care, and to determine whether or not cost-effectiveness differed between countries or according to the number of CHW-provided contacts a woman received.

## Methods

This was a planned secondary analysis of the clinical and cost data collected from the CLIP cRCTs in Karnataka state, India, Sindh province in Pakistan, and Maputo and Gaza provinces in Mozambique,^13^ based on a previously published protocol^14^ and reported in line with a Consolidated Health Economic Evaluation Reporting Standards checklist.^15^ (See online supplemental appendix 1 for the Statistical Analysis Plan.)

10.1136/bmjgh-2020-004123.supp1 Supplementary data

### Study population

The CLIP trials targeted pregnant women (15–49 years in India and Pakistan, and 12–49 years in Mozambique) across all intervention and control clusters (12 in each of India and Mozambique, and 20 in Pakistan). All women enrolled provided written consent. All were included in the economic analysis provided they had delivered by trial end and had data on the primary outcome of a composite of maternal and perinatal morbidity or mortality.

### CLIP intervention

The methods for the CLIP trials are described in detail elsewhere.^8 13^ Briefly, each of the cRCTs aimed to reduce all-cause maternal and perinatal morbidity and mortality by community-level initial triage and treatment of hypertensive women in their community, and timely transfer to facility. The intervention combined community engagement and pregnancy hypertension-focused home contacts by CHWs (ie, accredited social health activists and auxiliary nurse midwives (India), female health workers (Pakistan) and Agentes Polivalentes Elementares (Mozambique)). Community engagement meetings focused on pre-eclampsia awareness and education around birth preparedness and complication readiness, and included the pregnant women themselves, as well as their family members and community stakeholders. CHW-led home contacts were centred around the use of the PIERS (Pre-eclampsia Integrated Estimate of RiSk) On the Move (POM) mobile health application for risk stratification.^16^ The POM application helped CHWs to respond to emergency conditions, take women’s blood pressures, assess proteinuria (using dipsticks at the first and any subsequently hypertensive visits) and use pulse oximetry (in Pakistan and Mozambique). For hypertensive women, the POM application directed CHWs to administer oral methyldopa, intramuscular magnesium sulfate, or refer to a comprehensive emergency obstetric care facility depending on the particular circumstance. In the control group, women received routine antenatal and postnatal care. In all sites, postnatal care (as a standard) is rare, but antenatal care rates are high (76% receive at least four visits in Karnataka, 54% in Sindh and 51% in Mozambique). Antenatal care in all three sites typically takes place at local health or primary health centres on ‘antenatal care days’ and is provided by a mix of care providers. In both control and intervention arms, relevant demographic, care-seeking and clinical data were obtained at enrolment, through to 28 days post partum (newborn) and 6 weeks (mother), through regular cross-sectional household surveys (every 3–6 months) and, in India, with additional facility record review.

### Health systems

Each of the three healthcare systems has primary health centres, local inpatient facilities (combined with primary health centres in India), referral facilities and major referral centres. Each of the CLIP sites (Karnataka state in India, Sindh province in Pakistan, and Maputo and Gaza provinces in Mozambique) was a mix of rural and periurban settings and therefore does not represent the countries as a whole. In each setting, the CHWs have not been well integrated into any of the three formal health systems. The CLIP intervention was not a formal health system intervention.

### Costs

Within each country, detailed costs associated with the intervention were collected from annual budgets, receipts and the number of intervention-related activities performed. Costs were divided into five main categories: (1) CHW training, (2) health worker incentives for providing POM visits, (3) drug administration costs resulting from POM visits, (4) community engagement sessions (not including CHW staff costs), and (5) supplies; for details, see online supplemental table S2. Total cost of the intervention is the sum of these five categories. Discounting of costs was unnecessary due to the relatively short time period of the analysis (of less than 2 years). In the main analyses, we did not account for potential costs associated with differences in care seeking (antenatally or postnatally) between intervention and control, as these measures were broadly similar between arms.^7 9 10^ In Pakistan, we included out-of-pocket costs to women, gathered by pilot-tested focus group discussions, in a separate analysis (online supplemental table S2). In India and Mozambique, trial resources and logistics did not permit these group discussions, and only the above incremental intervention costs were available. We did not have data available for control arm costs (beyond those above for Pakistan) and, therefore, these were not included in the analyses. We did not account for the cost of trial surveillance in both arms, as a potential scale-up of this intervention would not incur these costs. Costs for each country were converted into US$ rates based on the average exchange rate during the trial (ie, India: US$1=INR60, Pakistan: US$1=PKR104.7, Mozambique: US$1=MZN64.67).

10.1136/bmjgh-2020-004123.supp2 Supplementary data

### Outcomes

The primary clinical endpoint was mortality for mothers and infants, including stillbirth and neonatal death. These rates were translated into years of life lost (YLL) based on country-specific WHO life expectancies.^17^ In contrast to the CLIP primary composite outcome, pregnancy-related morbidities for mothers and newborns were not included, as there are no validated years of life disabled or disabilities associated with many of these outcomes in any of the three countries.^18^ Cost-effectiveness was summarised by incremental cost-effectiveness ratios (ICERs), which are interpreted as the average incremental cost relative to 1 year of life saved. That is, the cost that a policymaker would have to pay to extend life by 1 year.

### Study perspective

The cost-effectiveness analyses were based on a programmatic perspective, comparing the incremental cost of implementing the intervention both for the overall intervention as delivered, and for various scenarios of POM-guided contacts delivered. This comparison assessed the cost to the health system of implementing the CLIP intervention at various levels but did not account for potential additional costs to women and their families (ie, patient costs were not included), other than in a secondary analysis in Pakistan.

### Data analyses

The primary economic model used for the base case analysis and probabilistic sensitivity analysis was a decision tree^19^ based on the following possible decision points: (1) arm of the trial; (2) number of POM contacts grouped into one of 0, 1–3, 4–7, or ≥8 (within intervention arm branch); (3) whether or not a POM-guided contact resulted in a referral to facility or use of either methyldopa or magnesium sulfate at any POM visit; and (4) outcome of pregnancy: maternal death, stillbirth, neonatal death or survival. See online supplemental figure S1 for details.

Within each country, the conditional probability at each branch was estimated directly from the CLIP trial surveillance and POM data. SEs for these probabilities were estimated and adjusted for the clustered structure of the data, and associated mean costs were estimated for each branch. Community engagement costs were assumed to be distributed evenly across the varying POM contact frequency groups. All other costs (CHW training and incentives, supplies, drugs) were distributed on a per contact basis, meaning branches on the tree corresponded to groups with more POM-guided contacts and associated costs, to appropriately estimate the associated implementation cost of each frequency (‘scenario’) of the intervention. In addition, we also computed the average cost per visit by summing the non-community engagement-related costs (CHW training, CHW incentives, drug administration and supplies) and dividing by the total number of visits.

We conducted a standard probabilistic sensitivity analysis^20^ to determine the uncertainty associated with our analyses. All analyses were carried out using R V.3.5.3 and RStudio interface. For this, probability and costs for each branch were simulated using the above parameters. Costs were assumed to have gamma distributions, outcomes were assumed to have beta distributions and the probability of each number of POM visits was drawn from a Dirichlet distribution (online supplemental table S3). These simulations were run 10 000 times for each country and the results summarised with 95% credible intervals (as the 2.5th and 97.5th percentiles of the 10 000 runs) for the YLL, cost and ICERs for each trial arm as well as at each intervention scenario. Results were also visualised using a cost-effectiveness plane (with points in the top, right corner considered to be cost-effective), and with willingness-to-pay curves and thresholds for each YLL averted (which shows for increasing cost to a decision-maker the likelihood of the intervention is cost-effective). Willingness-to-pay thresholds based on (1) one and three times the country-specific gross domestic product (GDP) per capita in 2016 (India=$1729 and $5188, Mozambique=$429 and $1287, Pakistan=$1368 and $4105) were used^21 22^ and (2) low and high-range estimates for country-level cost-effectiveness recently advocated (India=$115 and $770, Mozambique=$8 and $294, Pakistan=$87 and $669).^23^

### Patient and public involvement

The design of the CLIP trials was informed by extensive in-country qualitative work with communities, including focus groups with women, men and other decision-makers, and community leaders.^24–26^ There was an experienced patient representative on the Technical Advisory Group responsible for ongoing study surveillance (see the Acknowledgements section). Dissemination activities have been held with the participating communities in each country, and feedback about the intervention from women was very positive (eg, ‘you have brought the hospital to my home’).

## Results

Data from 61 988 pregnancies of 69 320 enrolled in CLIP (89.4%) were included in the CLIP economic analyses: 13 017 (88.1%) from India, 35 791 (90.8%) from Pakistan and 13 810 (91.3%) from Mozambique. The rates of maternal mortality, stillbirth and neonatal death did not differ between trial arms in any of the CLIP trials, but women receiving at least eight POM-guided contacts in India and Mozambique, and at least four contacts in Pakistan, had lower rates of each of these outcomes, with stillbirth (India and Pakistan) and neonatal mortality (Pakistan) being statistically significant, as previously reported.^7–10^ Outcome rates and trial intervention details can be found in table 1.

**Table 1:** Main trial outcomes and intervention

	India		Mozambique		Pakistan	
	Intervention n=6908	Control n=6109	Intervention n=6941	Control n=6239	Intervention n=18 441	Control n=17 350
**Trial outcomes**						
Composite maternal and perinatal outcome (%)	1252 (18.1)	1157 (18.9)	1246 (18)	1172 (18.8)	5373 (29.1)	4187 (24.1)
Maternal mortality (%)	7 (0.1)	9 (0.1)	15 (0.2)	7 (0.1)	55 (0.3)	51 (0.3)
Maternal morbidity (%)	371 (5.4)	325 (5.3)	735 (10.6)	690 (11.1)	2213 (12)	1728 (10.0)
Stillbirth (%)	191 (2.8)	156 (2.6)	196 (2.8)	162 (2.6)	935 (5.1)	951 (5.5)
Neonatal death (%)	179 (2.6%)	136 (2.2)	218 (3.1)	171 (2.7)	1011 (5.5)	962 (5.5)
Neonatal morbidity (%)	813 (11.8)	790 (12.9)	275 (4.0)	362 (5.8)	2375 (12.9)	1684 (9.7)
**Intervention**						
Community engagement sessions	1379 groups	–	4243 groups	1379 groups	1368 groups 16 691 CHW led	–
CHWs trained	148	–	79	–	223	–
POM-guided contacts (n)	57 562		26 145			54 782
POM-guided contacts per pregnancy	8.0 (3.0, 12.0)	–	4.0 (2.0, 6.0)	–	3.0 (2.0, 5.0)	–
0	770 (11.1%)	–	2796 (40.3%)	–	7905 (42.9%)	–
1–3	1268 (18.3%)	–	936 (13.5%)	–	2718 (14.7%)	–
4–7	1363 (19.7%)	–	1818 (26.2%)	–	6008 (32.5%)	–
≥8	3507 (50.8%)	–	1391 (20.0%)	–	1810 (9.8%)	–
Pregnancies given methyldopa (%)	60 (1.0)	–	28 (0.7)	–	93 (0.9)	–
Accepted (%)	51 (85.0)	–	19 (67.9)	–	92 (98.9)	–
Pregnancies given MgSO_4_ (%)	67 (1.1)	–	28 (0.7)	–	103 (1.0)	–
Accepted (%)	47 (70.5)	–	13 (46.4)	–	73 (70.9)	–
Pregnancies referred to facility (%)	505 (8.2)	–	263 (6.3)	–	487 (4.6)	–
Accepted (%)	401 (86.7)	–	158 (68.4)	–	305 (83.6)	–

CHW, community healthcare worker; MgSO_4_, magnesium sulfate; POM, PIERS (Pre-eclampsia Integrated Estimate of RiSk) On the Move.

Given the varying trial sizes, total implementation costs varied between countries; however, estimates of intervention costs per pregnancy were similar: US$13.0 in India, US$11.8 in Pakistan and US$15.7 in Mozambique. Table 2 shows that the majority of the costs in each country were related to POM-guided contacts and, therefore, costs were proportionately higher for women as they received an increasing number of contacts; the largest costs were for tablets and smartphones, and for the training of CHWs to deliver the intervention. In addition, costs in Pakistan were related to a much higher number of community engagement sessions compared with India and Mozambique. Other costs (eg, supplies) were similar on a per-pregnancy basis. Costs per POM visit were US$1.44, US$3.81 and US$2.65 in India, Mozambique and Pakistan, respectively. A complete detailed breakdown of costs can be found in online supplemental table S1.

**Table 2:** Summary costs for intervention by country

	India	Mozambique	Pakistan
Training of CHWs	12 755	53 205	79 398*
Incentives for delivering POM contacts	39 043	3097	
Methyldopa and MgSO_4_	1365	7163	4864
Community engagement	7048	9201	71 942
Supplies	29 811	45 383	60 949
Total	90 022	108 848	217 153
Total per pregnancy overall	13.0	15.7	11.8
0 POM-guided contact	1.01	1.35	3.91
1–3 POM-guided contacts	3.51	9.66	9.56
4–7 POM-guided contacts	8.06	22.10	17.48
≥8 POM-guided contacts	18.61	39.45	25.78
Cost of each POM visit†	1.44	3.81	2.65

All costs in US$.

*Unable to be disaggregated from available data.

†Sum of non-community engagements divided by total number of visits.

CHW, community healthcare worker; POM, PIERS (Pre-eclampsia Integrated Estimate of RiSk) On the Move.

**Figure 1:**
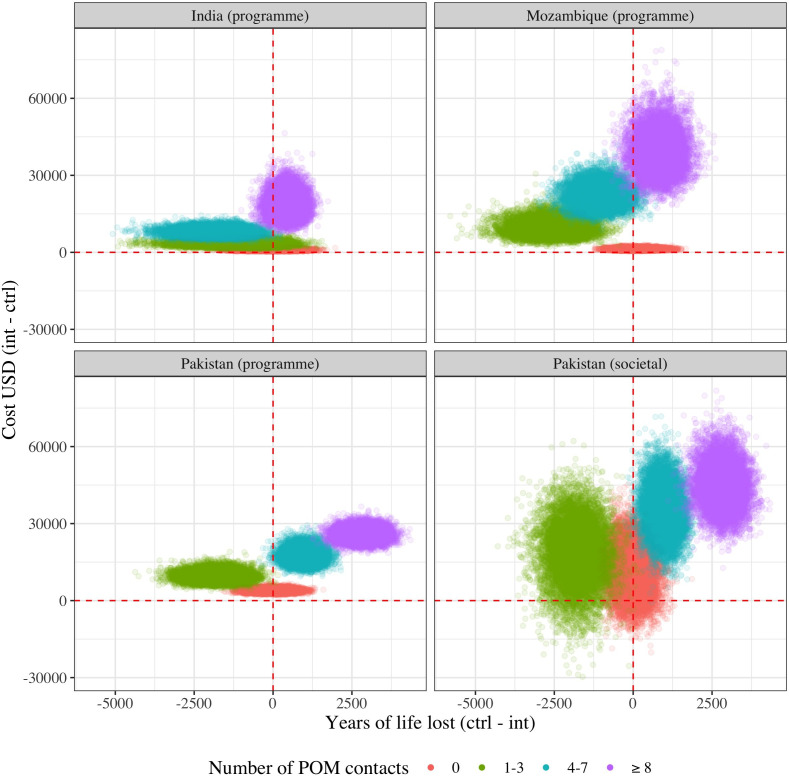
Probabilistic sensitivity analyses of cost-effectiveness by number of POM-guided contacts received. Points in the north-east quadrant are classified as cost-effective. All data are per 1000 pregnancies. ctrl, control; int, intervention; POM, PIERS (Pre-eclampsia Integrated Estimate of RiSk) On the Move.

In the base case analyses, the intervention was not found to be cost-effective in any of the three countries due to the lack of overall difference in maternal and perinatal mortality between arms (table 3). Similarly, in the probabilistic analyses, fewer than 50% of samples were cost-effective in Mozambique and India, and 66% were cost-effective in Pakistan (online supplemental table S4). However, when disaggregating the intervention based on POM-guided contact frequency group, the groups receiving ≥8 contacts (per protocol) showed cost-effectiveness in the base case in each country (ICERs=43.3, 48.7 and 9.1 for each of India, Mozambique and Pakistan, respectively); in addition, the four to seven contact group was cost-effective in Pakistan (ICER=17.8). The cost-effectiveness plane (figure 1) depicts the results from the probabilistic sensitivity analyses; in each country, >80% of the probabilistic samples were found to be cost-effective for the ≥8 contact frequency group.

**Table 3:** Cost and YLL summaries (95% credible interval from probabilistic sensitivity analyses) based on POM contacts received versus control arm

	India health system perspective				Mozambique health system perspective				Pakistan health system perspective				Pakistan societal perspective*				
	YLL	Incremental YLL	Incremental cost	ICER	YLL	YLL intervention–control	Incremental cost	ICER	YLL	YLL intervention–control	Incremental cost	ICER	YLL	Incremental YLL	Cost	Incremental cost	ICER
**Control**	3.36 (2.97, 3.78)		–	–	3.25 (2.65, 3.94)		–	–	7.63 (7.16, 8.11)		–	–	7.63 (7.16, 8.11)		132.33 (116.79, 145.04).		
**Intervention**	–		–	–	–		–	–	–		–	–	–			–	–
Overall	3.74 (2.68, 5.60)	0.38 (−0.82, 2.28)	13.0 (2.29, 23.6)	Intervention is dominated.†	3.67 (2.42, 5.65)	0.41 (−1.00, 2.43)	15.7 (1.40, 40.05)	Intervention is dominated.†	7.29 (5.23, 9.26)	−0.33 (−2.39, 1.60)	11.8 (3.70, 23.41)	12.94 (−188, 144)	7.29 (5.23, 9.26)	−0.34 (−2.38, 1.62)	155.75 (138.41, 174.89)	23.04 (0.41, 48.31)	67.80 (−316, 330)
0 POM-guided contact	3.41 (2.52, 4.43)	0.05 (−0.94, 1.10)	1.01 (0.49, 1.73)	Intervention is dominated.†	3.13 (2.69, 3.62)	−0.12 (−0.93, 0.64)	1.35 (0.71, 2.12)	11.25 (−45.6, 45.7)	7.65 (7.04, 8.30)	0.03 (−0.76, 0.82)	3.91 (2.77, 5.25)	Intervention is dominated.†	7.65 (7.04, 8.30)	0.03 (−0.77, 0.83)	143.39 (133.67, 152.53)	11.03 (0.00, 28.24)	Intervention is dominated.†
1–3 POM-guided contacts	4.53 (3.02, 6.42)	1.16 (−0.43, 3.07)	3.51 (1.84, 5.77)	Intervention is dominated.†	5.85 (4.69, 7.12)	2.60 (1.28, 4.03)	9.66 (4.80, 16.18)	Intervention is dominated.†	9.42 (8.46, 10.43)	1.80 (0.72, 2.94)	9.56 (6.21, 13.59)	Intervention is dominated.†	9.42 (8.46, 10.43)	1.80 (0.72, 2.92)	150.87 (131.70,165.68)	18.51 (0.00, 40.21)	Intervention is dominated.†
4–7 POM-guided contacts	5.23 (4.03, 6.68)	1.87 (0.58 3.34)	8.06 (5.57, 11.02)	Intervention is dominated.†	4.34 (3.60, 5.16)	1.09 (0.08, 2.09)	22.10 (14.97, 30.52)	Intervention is dominated.†	6.65 (6.21, 7.11)	−0.98 (−1.63, −0.31)	17.48 (12.72, 22.96)	17.84 (9.68, 51.0)	6.65 (6.21, 7.11)	−0.98 (−1.63, −0.30)	167.89 (156.56, 176.35)	35.54 (18.73, 53.70)	36.26 (15.10, 114)
≥8 POM-guided contacts	2.94 (2.46, 3.45)	−0.43 (−1.07, 0.22)	18.60 (11.30, 28.2)	43.28 (−359, 426)	2.44 (1.89, 3.08)	−0.81 (−1.70, 0.07)	39.45 (26.15, 55.32)	48.70 (−155, 323)	4.80 (4.15, 5.48)	−2.83 (−3.64, −1.99)	25.78 (21.49, 30.32)	9.11 (6.64, 13.20)	4.80 (4.15, 5.48)	−2.83 (−3.64, −1.99)	177.22 (169.14, 183.17)	44.87 (29.61, 61.28)	15.86 (9.65, 25.7)

All intervals are 95% credible intervals based on the probabilistic sensitivity analysis.

All data are per pregnancy, comparisons are to the control arm and all costs are in US$.

*Includes health system utilisation costs from care seeking reported by focus group.

†Indicates that the intervention has no benefit in years of life lost.

ICER, incremental cost-effectiveness ratio; POM, PIERS (Pre-eclampsia Integrated Estimate of RiSk) On the Move; YLL, years of life lost.

**Figure 2:**
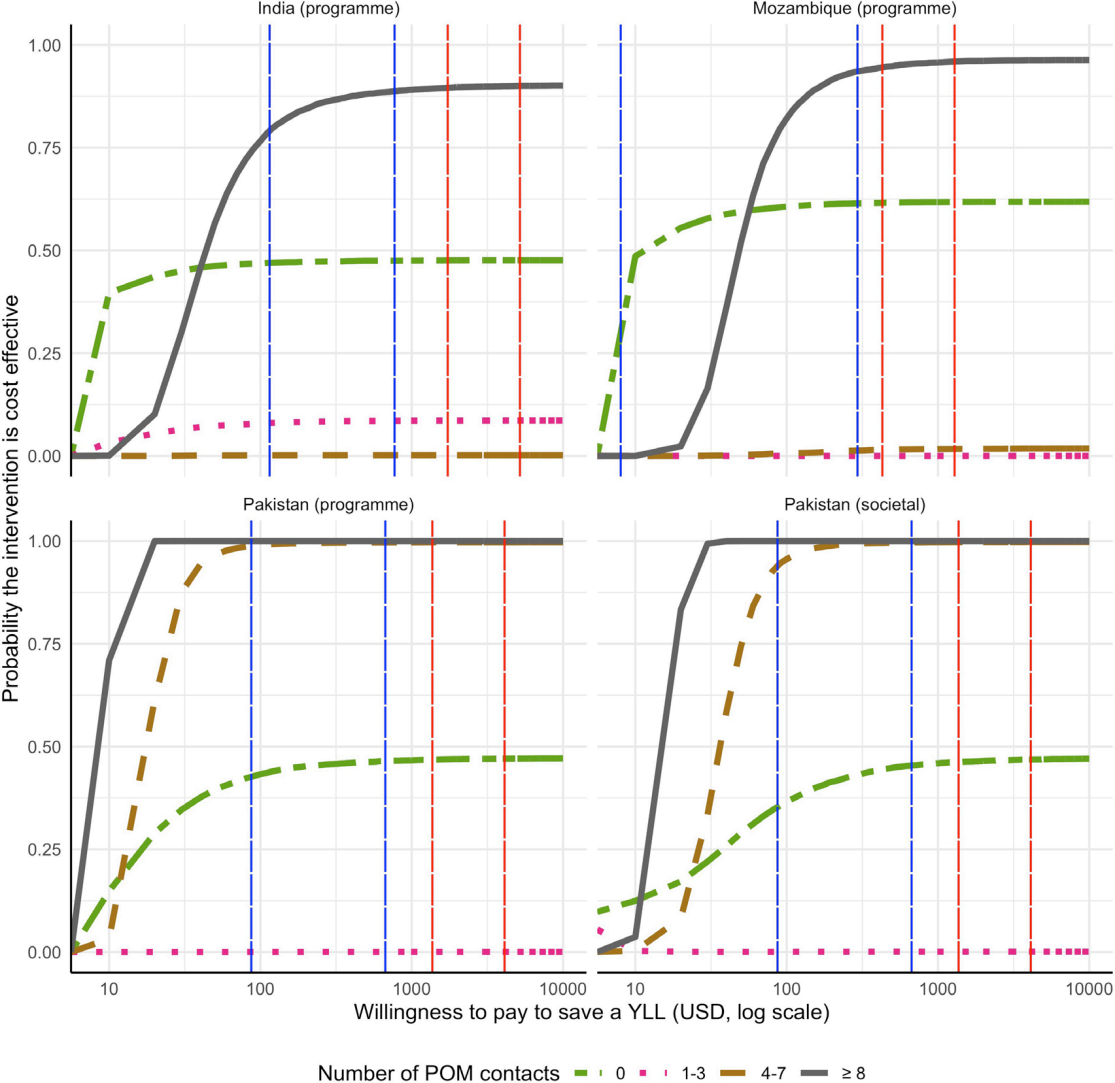
Cost-effectiveness acceptability curves by number of POM-guided contacts received: probability that the intervention is cost-effective as a function of a decision-maker’s willingness to pay to save 1 year of life lost. The vertical red line represents country-specific willingness-to-pay thresholds based on 1× and 3× the gross domestic product (GDP) per capita. The vertical blue lines represent the low and high points of country-specific willingness-to-pay ranges from Woods *et al* 23 POM, PIERS (Pre-eclampsia Integrated Estimate of RiSk) On the Move; YLL, years of life lost.

The cost-effectiveness acceptability curves (figure 2) show the probability of the intervention (for various scenarios) being cost-effective as a function of a decision-maker’s willingness to pay to save 1 YLL (log scale). In each country, if a decision-maker was willing to pay three times the GDP per capita (red dashed line), the ≥8 contact scenario (black solid line) has at least 90% certainty of cost-effectiveness. In Pakistan, at US$400/YLL, the probability of cost-effectiveness approaches near certainty (100%) for both the ≥8 and 4–7 contact scenarios (red dashed line). Similar findings in each country were found when the willingness-to-pay threshold was reduced to one times the GDP per capita and when willingness-to-pay thresholds were lowered to previously published country-specific recommendations.

In Pakistan, the secondary analyses focusing on a societal perspective (including out-of-pocket costs to women and their families) yielded similar results to the primary analysis, although with slightly higher costs associated with each intervention contact frequency group, corresponding to slight increases in healthcare utilisation in the intervention arms. Details are in table 3.

## Discussion

### Principal findings

This study assessed the cost-effectiveness for YLL, from the health system perspective, of the CLIP interventions in India, Pakistan and Mozambique. Using the Medical Research Council Process Evaluation of Complex Interventions guidance^27^ we observed that the incomplete implementation process overall resulted in no significant statistical difference in outcomes between trial arms, and, therefore, a lack of cost-effectiveness. This was despite contextual adaptations of the generic protocol to create bespoke interventions for each country. However, once fidelity with the published protocol and implementation plan were achieved in terms of both dose and reach, maternal and perinatal mortality was significantly lower in women who received at least eight POM-guided contacts (four in Pakistan), and that in those scenarios the intervention had a high probability of cost-effectiveness in each country. The cost of the intervention was remarkably similar between the three countries on a per-pregnancy basis (≈US$12–16). In addition, in Pakistan, although incremental costs were roughly twice as large, there were similar findings of cost-effectiveness when including women’s families’ opportunity (out-of-pocket) costs for health system resource utilisation.

### Interpretation

Several recent reviews have summarised the current evidence on CHW-led initiatives,^28–30^ and many previous studies have identified cost-effectiveness for a variety of CHW-led interventions in reproductive, maternal, neonatal and child health,^28 29^ with further evidence supporting the use of integrated ‘packages’ of interventions.^31^ Despite this substantial body of information, many maternal health studies still do not include a health economic component,^14^ and those focused on pre-eclampsia are based on diagnostic and clinical interventions, such as the use of magnesium sulfate.^32 33^ To our knowledge, this health economic analysis is the first to evaluate community-level interventions for pregnancy hypertension.

The observed differences in clinical and cost consequence outcomes are unlikely to be due to behaviour change as the individual trial analyses did not identify increases in care seeking in the intervention (vs control) clusters.^7–10^ Therefore, our results are broadly consistent with prior findings, although requiring a frequency of contacts from CHWs that matches current WHO guidance. Indeed, the consistently observed decrease in maternal and perinatal mortality, and increase in cost-effectiveness, with at least eight POM-guided contacts provides external validation of the recent WHO recommendations for at least eight antenatal care contacts.^34^ The contextual moderators that influenced the fidelity of implementation probably included the numbers of deployed CHWs, competing demands on CHW time (eg, ‘immunisation months’), the variable resilience of the emergency transport system once women at risk were identified and the timeliness and quality of the clinical responses as women ascended the referral system.

Although the overall per-pregnancy costs were similar between countries, there was discrepancy in the cost of delivering at least eight POM-guided contacts, which was largely driven by differences in CHW training and incentive costs per pregnancy. When considering at-scale implementation of interventions such as CLIP that require a high number of contacts to be effective, these cost differences would be important to consider, as CHW salaries and relative affordability differ between LMICs.^35 36^ That said, the relatively low cost in each country supports CHWs carrying out home-based visits during pregnancy in LMICs where interventions are urgently needed to reduce the burden of maternal and perinatal mortality.

The out-of-pocket costs to women in Pakistan were much higher than those based on the incremental cost of the intervention to the health system, and reveal the significant burden placed on families in order to seek appropriate routine and emergency care, obtain transport to facility and be admitted to health facilities for delivery. These costs reflect productivity losses due to pregnancy and pregnancy complications. As most families do not have sufficient savings, without societal safety nets, large pregnancy-related expenditures are a barrier for upward social mobility.^37^ Given the underlying differences in healthcare systems, the generalisation of these secondary societal results to India, Mozambique or other LMIC settings should be interpreted cautiously.

### Strengths

Our study has several strengths. First, we prospectively collected both cost and outcome data within our clinical trial.^11^ Second, we implemented our intervention across three diverse settings in Africa and South Asia, and with a large sample size of >60 000 pregnant women overall. Therefore, we believe that our findings are generalisable to other LMIC settings. Further, the details of our budget allow for clear understanding of the cost of scaling any one or all components of the intervention. In the case of Pakistan, we found similar cost-effectiveness at increased POM contacts (but not for the overall intervention) when including data on out-of-pocket costs to women and their families, and, therefore, providing a broader societal perspective on the cost-effectiveness.

We were further able to corroborate our main findings by including data on out-of-pocket costs to women and their families, and, therefore, providing a broader societal perspective on the cost-effectiveness.

### Limitations

The main challenge associated with our economic analyses is that we were unable to use disability-adjusted life-years (DALY) that include maternal and perinatal morbidity as an outcome measure. This is due to a lack of reliable DALYs for the collected morbidities in each of India, Pakistan and Mozambique. Given that we consistently observed similar results in maternal morbidity (no effect overall, decrease with at least eight POM contacts) to mortality^8^ it is probable that the cost-effectiveness results seen here would be similar if this could be included.

On the other hand, survived neonatal morbidity was in some cases increased in the ≥8 POM contact group, suggesting a trade-off between reduced mortality in this group and increased survivable morbidity in neonates, which would require further exploration to determine severity as well as the values of women, healthcare providers and their communities. Therefore, the inclusion of these data may have reduced the estimates of cost-effectiveness seen here.

Furthermore, given the scale of the trial and complexities of the health systems, reliable data on the out-of-pocket costs to women and health system costs associated with being referred were unavailable in India and Mozambique; this limits our conclusions to that of a health system perspective, rather than the preferred ‘societal’ approach in these two countries.^38^ Further, the lack of feasibility for collecting of out-of-pocket costs prevented us from doing an individual patient-level cost-effectiveness analysis. In Pakistan, estimates of family opportunity cost data were available from focus groups, and cost-effectiveness results were broadly similar to the programmatic perspective. These analyses relied on extrapolating these estimates to the larger trial population and therefore should be interpreted cautiously.

In addition, we did not adjust costs for differences in routine antenatal care seeking between arms as these measures and the associated cost were not available. Despite this, these measures were broadly similar between trial arms, and, therefore, we do not anticipate that this influenced the results.^7 9 10^ The CLIP trials relied on leveraging existing CHWs in each of the sites, and therefore we did not measure costs associated with increasing the number of CHWs, only the training and materials associated with the CLIP intervention. Therefore, there would therefore be higher costs in settings where CHWs are understaffed or not employed.

Finally, the decision tree model used could not adjust for differences between women who received a low and high number of POM visits that would have an impact on mortality, which may introduce a bias due to possible confounding. That being said, in the analyses of the primary trial data, adjustment for such measured differences did not affect the association between increased visits and reduced outcomes.^7–10^

### Meaning

Depending on a decision-maker’s willingness to pay, having CHWs deliver home-based interventions at WHO-recommended frequency thresholds is probably a cost-effective method for reducing maternal and perinatal mortality. Reaching these contact frequencies for all women may be challenging without resources beyond those available in CLIP. The similarity in the cost of CHW visits and per-pregnancy trial costs overall between countries points to generalisability to other LMICs.

### Future work

As further emphasis is placed on increasing the number of antenatal contacts in LMICs, future studies should continue to assess the cost-effectiveness of delivering these contacts, and integrated interventions by CHWs. Our data suggest that CHW-based interventions can be effective and cost-effective if a sufficient number of workers are available to deliver them at the required frequency. Future studies should estimate both health system and societal perspective costs in settings where families bare the cost of facility-based care. In addition, there is a need for continued development of ‘years-of-life-disabled’ metrics for pregnancy-specific morbidities so that trials can better estimate the economic impact of their interventions.

## Conclusion

While the CLIP intervention as implemented was not cost-effective at reducing YLL in any of the three countries, when implemented with at least eight POM-guided contacts per pregnancy, there was both a reduction in YLL and a high probability of cost-effectiveness in each of the three CLIP countries. This supports the eight antenatal care contacts model advocated by the WHO.

## Data Availability

Data are available upon request. As per the Statistical Analysis Plan (SAP), following publication of the primary CLIP manuscripts, and individual participant data meta-analysis, the data will be freely available to academically active entities (eg, universities, NGOs, multilaterals), with the CLIP principal investigator (PvD) or named delegate as a named coinvestigator, for the purposes of pregnancy-related research and within the limits of the informed consent obtained. Access will be through the CLIP Trials Data Access Committee*, contacted at ‘PRE-EMPT@cw.bc.ca’, as referenced on our website at ‘https://PRE-EMPT.obgyn.ubc.ca’. A copy of the data will also be deposited with our funder, the Bill & Melinda Gates Foundation, in the HBGDki repository.
