# Supplementary material for: Economic and cost-effectiveness analysis of the Community-Level Interventions for Pre-eclampsia (CLIP) trials in India, Pakistan and Mozambique
Source: BMJ Glob Health. 2021 May 24;6(5):e004123. doi: 10.1136/bmjgh-2020-004123 (PMC8149358; doi:10.1136/bmjgh-2020-004123)
Supplement: Supplementary data [file bmjgh-2020-004123supp001.pdf]

Document Title:	**Statistical analysis plan for CLIP Pilot and Definitive Trials**
Version:	1.7
Date:	Drafted 16 October 2015, amended last March 1, 2017
Authors:	Peter von Dadelszen, Tang Lee, Joel Singer, Hubert Wong, Sumedha Sharma, Beth Payne, Marianne Vidler, Zulfiqar Bhutta, Laura A Magee
Protocol Number:	ClinicalTrials.gov identifier NCT01911494
Study Title:	Community level interventions for pre-eclampsia (CLIP) Trials
Principal Investigator:	Peter von Dadelszen

This document has been reviewed and approved by:

Statistician<br>*Tang Lee*		March 1, 2017
Program co-ordinator<br>Sumedha Sharma		March 1, 2017
Principal Investigator<br>Peter von Dadelszen		March 1, 2017

Table of Contents

1. Introduction 3
1.1 Study Description 3
1.1.1 Update to CLIP Nigeria 3
1.2 Trial Objectives 4
Primary Objectives 4
Secondary Objectives 4
1.3 Sample size calculations 4
2.0 Data summaries and analyses 5
2.1 Trial information and patient disposition 6
2.1.1. Consort diagram 6
2.1.2. Baseline and demographic variables 6
2.2 Primary analysis 8
2.2.1 Pilot Trials 8
2.2.2 Definitive Trials 8
2.2.2.1 Definition of the primary outcome 9
2.2 2.2 Calculation of the primary outcome 10
2.2.2.3 Analysis of the primary outcome 10
2.2.3 Sensitivity analyses 11
2.2.3.1 Completed pregnancies with postpartum follow-up 11
2.2.3.2 Complete postpartum follow-up (for all components of primary outcome) 11
2.2.3.3 Women whose EDD+3 weeks falls within the trial timeline 11
2.2.3.4 Women whose EDD+3 weeks plus 42 day follow up period falls within the trial timeline 11
2.2.3.5 Without adjustment 11
2.2.3.6 For women in India 11
2.2.3.7 Evaluating 'on treatment' effect: 11
2.3. Application of the intervention 11
2.4 Secondary Analyses 12
2.4.1 Secondary outcomes 12
2.4.2 Other Outcomes 13
2.4.3 Other Planned secondary analysis 13
2.5 Interim Analysis 15
2.6 Safety Data 15
2.6.1 Definition of Adverse Events (AEs) 15

2.6.2 Definition of Serious Adverse Events (SAEs) 16
2.7 Extraction of per-pregnancy data 16
2.8 Individual patient data meta-analysis (IPD) 16
3.0 Additional Methodological Details 17
3.1 Statistical Tests 17
3.2 Software to be Used 17
4. Appendix 18
4.1 CLIP country trials Sample Size Calculations 18
4.2 CLIP IPD Meta-analysis Power Calculations 20
4.3 Withdrawal 20
4.4 The Geo-Temporal analysis 20
4.5 Health Economics – Plan of analysis 21

Appended in the 'SAP Tables' document are the following

Table 1 Design of the Pilot Trial

Table 2 Design of the Definitive Trial

Table 3 Data fields used in the analysis of baseline and demographic variables

Table 4 Baseline outcome variables collected, by country

Table 5Decision rule for POM acceptance of referral

Table 6 Data fields used in the analysis of POM acceptance of referral

Table 7 Data fields used in the analysis of primary outcome

Table 8 Data fields used in the analysis of secondary outcomes

Table 9 Data fields used in the analysis of other outcomes

Table 10 Data fields used in the analysis of adverse events

1. Introduction

1.1 Study Description

A cluster randomised trial designed to evaluate the implementation of community-level evidence-based care aimed at reducing pre-eclampsia-related maternal and perinatal mortality and major morbidity.

The CLIP intervention consists of the following three components:

(i) **Community engagement** including community leaders, the women of the communities themselves, and their mothers, husbands, and mothers-in-law, regarding pre-eclampsia, its origins, symptoms, signs, and potential consequences, pre-permissions for maternal transport, and microfinancing activities around transport and treatment costs;

(ii) Provision of hypertensive disorders of pregnancy (HDP) oriented antenatal care through **CLIP visits** and use of CLIP PIERS On the Move (POM) tool for risk stratification. Community-based health care providers (cHCPs) will assess pregnant women with a target frequency of every 4 weeks at a minimum, and according to protocol. These visits can occur in the home or primary health centre (PHC) as both are considered part of the community for the purpose of the CLIP Trial. They will be trained to enquire about women's symptoms (using country-specific pictograms), take women's blood pressure (BP) (using systolic blood pressure (sBP), check urine for protein using dipstick and measure SpO2 (in Pakistan and Mozambique) on the first visit or on any subsequent visits for sBP ≥140 mmHg. This will inform the diagnosis and risk assessment of women with pre-eclampsia.

(iii) Use of the **CLIP 'package of care' for women with a CLIP 'trigger'** as indicated on the POM tool (i.e., oral antihypertensive therapy when indicated, intramuscular (i.m.) MgSO4 when indicated; and appropriate referral to an comprehensive emergency obstetric care (CEmOC) facility when indicated).

Facility enhancement and health worker training will be conducted in both intervention and control clusters. This activity is designed to enhance skills and promote evidence-based pre-eclampsia identification and management so that women sent to facility receive quality care.

The trial was designed to occur in two phases - a Pilot phase and a Definitive phase. The Pilot phase was designed to show that the intervention was feasible, and the data for the pilot phase form part of the definitive phase analysis. As shown in Tables 1 and 2, the Pilot trial involved four clusters per country, and the number of clusters was expanded in the Definitive phase. Surveillance data from the Pilot Trial was not analysed or reviewed and no outcomes were reviewed until after the end of the Definitive Trial. The trial protocol has been registered on clinicaltrials.gov with trial identifier NCT01911494.

1.1.1 Update to CLIP Nigeria

The CLIP Nigeria trial was originally designed to be undertaken in Ogun State, Nigeria as a pilot and then definitive cRCT, as in the three other CLIP countries. However, challenges with data collection and data entry prompted the decision to change the research design in Nigeria to one of process evaluation. To complement the effectiveness trials in Mozambique, India, and Pakistan, the process evaluation in Nigeria will serve to assure policy makers in Nigeria that the CLIP intervention can be undertaken successfully and is supported by both the community at large and health workers at various levels. The process evaluation in Nigeria will involve data collected electronically on the POM mobile health device. Excluded from analysis will be data that were initially collected on paper and then entered electronically,

as data entry was unreliable; these data involve the baseline household survey (feasibility study pre-pilot trial) and both pregnancy registration and trial surveillance (as part of the pilot and definitive trials).

1.2 Trial Objectives

Primary Objectives

- Pilot Trial (for each of Nigeria, Pakistan and India)– To implement the use of the CLIP 'package of care' and to observe at a minimum 50% acceptance of referral [urgent or non-urgent, to a facility able to provide CEmOC (Comprehensive Emergency Obstetric Care)] in women with a CLIP trigger
- Definitive Trial (for each of Mozambique, Pakistan and India) – To reduce pre-eclampsia-related, and all-cause, maternal and perinatal mortality and major morbidity by 20% or more in intervention clusters compared with control clusters.
- IPD meta-analysis (combining results from Mozambique, Pakistan and India)-
    - Maternal mortality & morbidity (primary outcome for the IPD meta-analysis): reduction by ≥ 20%
    - Maternal and fetal/neonatal mortality & morbidity: reduction by ≥ 20%
    - Maternal mortality: reduction by ≥30%

More details are in section 2.8 and power calculations for the IPD meta-analysis are in Appendix section 4.3

Secondary Objectives

Definitive Trial – (1) To measure the impact of the CLIP intervention on birth preparedness and complication readiness, the proportion of women who present for care at a CEmOC, and the proportion of women who deliver in facility.

1.3 Sample size calculations

Each country is independently powered to assess the chosen primary outcome. The data upon which the estimates have been made are routinely at the conservative end of the published or available range, and were generally provided by the site investigators. Sample size calculation for the primary outcome can be seen in Appendix 4.1. However, these calculations were revisited as planned originally, after data from the CLIP Feasibility Study and CLIP Pilot maternal and perinatal morbidity and mortality data were collected.

This review conducted independently by the Statistical Team provided insight that neonatal mortality rate in baseline varied greatly across clusters. At this point, it was also suggested by the Statistical team that adding more clusters would increase statistical power- however, this was not deemed feasible. A suggestion was then made by the statistical team to add neonatal mortality rate as a cluster level adjustment factor. More details on adjustment factors are in Section 2.1.2.

Bone JN, *et al. BMJ Global Health* 2021; 6:e004123. doi: 10.1136/bmjgh-2020-004123

2.0 Data summaries and analyses

Participants will be all pregnant women who consent to CLIP data collection, are of reproductive age (i.e., 15-49 yr in Pakistan and India, and 12-49 in Mozambique), and are identified by the community health care providers (cHCPs) assigned to their community. Data will be analysed and reported separately for each country; an individual patient level (IPD) meta-analysis combining data from all countries will be conducted following completion of all individual trials (see Section 2.7). All data will be presented by intervention and control clusters unless otherwise specified, and for all countries combined. Section 2.3.2 outlines subgroup analyses that will include within-country analysis.

2.1 Trial information and patient disposition

2.1.1. Consort diagram

The following information will be presented:

- N women enrolled, as defined by consent to participate in trial surveillance.
- N women who withdrew
- N women who were lost to follow-up
- N women who were still on follow-up at the end of the trial
- N women who were followed up postpartum

These categories will be defined as follows:

- **Withdrawal** (from trial surveillance) **-** captured from the withdrawal log on REDCap and Monthly Reports, identifying women who have withdrawn from data surveillance. This is further described in appendix 4.3
- **Lost to follow-up** - women without trial surveillance, who were > 6 weeks postpartum more than one surveillance cycle from the end of the trial
- **Still on follow-up**
    - Antepartum women, **or**
    - Women, without postpartum trial surveillance, who were ≤ 6 weeks postpartum within one surveillance cycle† from the end of the trial, **or**
- **Followed-up** (i.e., have postpartum trial surveillance)†
  † *One surveillance cycle has been defined as 3 months (as in Pakistan), with 1 added to err on the side of caution.*

2.1.2. Baseline and demographic variables

Summary statistics (mean, median, standard deviation, minimum, maximum and inter-quartile range for continuous variables; the number and percentage of patients with various levels of categorical variables) will be calculated for each country, for:

- Each of intervention and control clusters; and
- Within each of intervention and control clusters, for women who were (i) lost to follow-up, (ii) still on follow-up at the end of the trial, and (iii) followed up. This analysis will enable us to see how women who were followed up may have differed from women who were not.

Adjustment will be undertaken to improve precision. Adjustment for individual level variables is likely to have less impact on the results. However, we will also include these variables to maintain consistency with how cluster level adjustment variables are being handled. The planned **adjustment variables** are at both individual and cluster-levels, in addition to any other characteristics that differ between groups at the individual and cluster level, as follows:

Individual level* (N=3): maternal age(as a continuous variable),* ***parity****(nulliparous/multiparous***),** *and and* ***maternal basic education (****defined in Mozambique as attainment of Grade 5 or above, and in Pakistan as completion of 5 years or more of schooling and in India as 8 years or more of schooling. There is no formal*

*definition of literacy that can be applied across all settings, however, primary schooling was defined based on the country specific information provided by the site investigators)***.**

The following were considered by the committee and rejected for use:

- 'Distance from facility' (defined as primary health centre, PHC) - Average distance would have to be modelled 'as the crow flies' (i.e., using straight lines from home to PHC) for India and Pakistan because there, more precise travel distances based on the actual road network cannot be calculated because of the lack of accessibility of street datasets.
- Gestational age at enrolment - Women in intervention clusters were exposed to the intervention (community engagement) and may have booked earlier for antenatal care. By adjusting for gestational age at enrolment, we may adjust for treatment effect. It is also assumed that if women (in intervention clusters) enrol in the trial earlier, we may be able to capture greater number of outcomes such as miscarriages.
- Maternal past medical history of hypertension or seizures/epilepsy

Cluster level* (N=2): population density(population of cluster/unit size of cluster***) and the 'baseline' neonatal mortality rate (***from the baseline household survey)*

The following were considered by rejected for use:

- The poverty index as a measure of SES is no longer being considered, as BPL card in India is not considered a reliable measure of SES.
- cHCP density is no longer being considered.

Although baseline neonatal mortality rates were not considered in the design of either the original pilot or definitive trials, these rates were found to vary substantially across clusters in the analysis of the data gathered in the Feasibility Study. It was felt that their inclusion as an adjustment variable in the final analysis would substantially improve the power to detect a treatment effect. Without knowledge of the pilot trial results, the CLIP trial steering group elected to include based neonatal mortality from the pilot trial as an adjustment in the primary analysis.

2.2 Primary analysis

All analyses would be intention to treat. All statistical tests will be two-sided, with significance levels of < 0.05 for the primary outcome, and <0.01 for secondary and other outcomes. Comparisons will be by means of risk ratios and mean differences with 95% confidence intervals for each site.

2.2.1 Pilot Trials

At the end of the specified Pilot period per country (with the exception of Mozambique), the Piers On the Move data from intervention clusters was analysed for completed pregnancies to calculate referral acceptance. Surveillance data was not reviewed for intervention and control clusters except by statistical teams.

Referral acceptance was determined based on confirmation at a follow-up visit that the woman received treatment and/or went to facility. The concordance between these initial and follow-up compliance responses will be used to impute compliance for women for whom there was no follow-up visit. A minimum 50% acceptance of referral [urgent or non-urgent, to a facility able to provide CEmOC (Comprehensive Emergency Obstetric Care)] in women with a CLIP trigger was used to assess the transition of the study site from the Pilot to Definitive Trial.

These process data were shared with the CLIP Steering Committee to assess the progression of country from the Pilot to Definitive Trial. No outcome data from either intervention and control arms would be reviewed until after the end of the Definitive Trial. The pregnancies included in the Pilot trial were carried over into the Definitive Trial and will be analysed for primary outcome at the end of the Definitive Trial.

2.2.2 Definitive Trials

All analyses will be based on intention-to-treat principles. The primary outcome is the proportion of pregnancies resulting in at least one of maternal death, stillbirth, neonatal death, or severe morbidity in the mother or child.

Multiple imputation by chained equations (MICE) will be used to fill missing values, for both explanatory and outcome variables, before regression modeling of outcomes. Imputation will be undertaken for the composite primary outcome and then for each category of the primary outcome (i.e. maternal mortality, maternal morbidity, neonatal mortality and neonatal morbidity). This assumes that missing values are missing completely at random, that is, there are no systematic differences between the missing values and the observed values. Imputation will be repeated at least ten times, and outcome rates will be modeled in each of the derived dataset.

A hierarchical logistic regression model will be used to take into account the clustering of women within the units of randomisation. The model will adjust for key determinants of outcome, pre-specified by the investigators, at both the individual and cluster level.

Imputation will be based on adjustment variables (both individual and cluster level) and will include a term to account for any potential interaction between intervention clusters and time of enrolment (i.e. recruitment within the first 12 months of the trial vs. last 12 months).

2.2.2.1 Definition of the primary outcome

Maternal death (defined as the number of deaths during pregnancy or within 42 days of delivery (or the last postpartum contact day if contact not maintained to 42 days postpartum) / 1,000 identified pregnancies)

Maternal morbidity (defined as the number of women with one or more life-threatening complications of pregnancy during pregnancy or within 42 days of delivery or last postpartum contact day if contact not maintained to 42 days) / 1,000 identified pregnancies). These are the serious end-organ complications of pre-eclampsia, other major causes of maternal mortality, or life-saving interventions related to one of the aforementioned:

Serious end-organ complication of pre-eclampsia:

- Eclampsia: occurrence of generalised convulsions during pregnancy, labour or within 42 days of delivery in the absence of epilepsy or another condition predisposing to convulsions
- Stroke: hemiparesis and/or blindness developed during pregnancy or in the 42 days postpartum lasting greater than 48 hours
- Coma: prolonged unconsciousness ≥12 hours
- Antepartum haemorrhage: vaginal bleeding ≥ 15 mL with or without pain before the onset of labour
- Disseminated intravascular coagulation (DIC): abnormal bleeding from mucosa(mouth and/or ears)

Other major causes of maternal mortality:

- Obstetric sepsis: In the community, defined as fever and one of: abdominal/uterine tenderness, foul smelling vaginal discharge/lochia, productive cough and shortness of breath, dysuria or flank pain, headache and neck stiffness. In the facility, defined as presence of fever (>38°C), a confirmed or suspected infection (e.g., chorioamnionitis, septic abortion, endometritis, pneumonia) and at least one of the following: heart rate >90/min, respiratory rate >20/min, leukopoenia (total leukocyte count [TLC] <4 x 10^9/L) or leukocytosis (TLC >12 x 10^9/L)
- Vesicovaginal or rectovaginal fistula: continuous loss of urine and/or faeces after delivery

Life-saving interventions:

- Cardiopulmonary resuscitation: a set of emergency procedures including chest compressions and lung ventilation applied in cardiac arrest victims
- Dialysis: haemodialysis and/or peritoneal dialysis
- Mechanical ventilation (other than for Caesarean delivery): intubation and ventilation not related to anaesthesia
- Blood transfusion: ≥1 unit of any type of blood product
- Interventions for major postpartum haemorrhage: brace sutures, external and internal uterine compression, anti-shock garment use, internal iliac artery ligation and/or hysterectomy with or without transfusion

Perinatal & late neonatal death (defined as stillbirth [≥20^{+0} weeks and/or ≥500g in weight], early

Bone JN, *et al. BMJ Global Health* 2021; 6:e004123. doi: 10.1136/bmjgh-2020-004123

neonatal mortality [d 0-7 of postnatal life] and late neonatal mortality [d 8-28 of postnatal life] / 1,000 identified pregnancies)

Neonatal morbidity (defined as occurrence of a primary neonatal morbidity during d 0-28 of postnatal life / 1,000 identified pregnancies). The following are the primary neonatal morbidities:

- Feeding difficulty: Including inability to suckle normally or latch on to the mother's breast to feed even if the mother's milk is not let down
- Breathing difficulty: Including grunting and in-drawing of the abdomen under the ribs
- Seizure: Occurrence of any seizure event (fits)
- Lethargy: Baby not appearing normally wakeful after activities such as feeding or sleeping
- Coma: Not medically induced period of unconsciousness of any length
- Hypothermia: Cold to touch
- Umbilical cord infection: Characterized by discharge from and redness around the umbilical stump
- Skin infection: Any appearance of abnormally red, black, swollen and blistered skin with pus
- Bleeding: From anywhere
- Jaundice: Yellow skin and eyes
- Central nervous system related morbidity: Abnormal amount of vomiting as defined by the parents or caregiver with bulging or sunken fontanelle

2.2 2.2 Calculation of the primary outcome

All enrolled pregnancies, with the exception of those who withdrew consent from the trial, will be included in the analysis of primary outcome. For women who were 'lost to follow up' or 'still on follow up' at the end of the trial, the primary composite outcome data will be imputed using a mixed imputation based that takes into account the risk associated with each woman depending on her personal characteristics (from pregnancy registration), cluster characteristics, and time of enrollment relative to the beginning of the trial.

2.2.2.3 Analysis of the primary outcome

The data fields used in the analysis of primary outcome composite is table 7, and primary outcome is calculated from the components using the following rules:

Primary outcome occurred: If ANY of the individual components were answered 'yes' (even if some components were missing answers). Define which components were missing, and the N (%) women who had one, two, three, etc, of the components missing.

Primary outcome did not occur: If none of the components were answered 'yes', and, ANY of the components was answered 'no' (even if some components were missing answers). Define which components were missing, and the N (%) women who had one, two, three, etc, of the components missing. Our assumptions can be checked by comparing patterns of responses among women who had complete and incomplete response sets.

Primary outcome is missing: If NONE of the components was completed as 'yes' or 'no' (only 'unknown' and missing were recorded). This women will be included in the imputation.

Bone JN, *et al. BMJ Global Health* 2021; 6:e004123. doi: 10.1136/bmjgh-2020-004123

Denominator for the primary outcome rate will be all women included in the analysis (primary outcome occurred, primary outcome did not occur, and primary outcome missing).

These rules will also be used for the calculation of all other composite variables. Section 2.3.3 shows primary outcome and its components.

2.2.3 Sensitivity analyses

2.2.3.1 Completed pregnancies with postpartum follow-up:*This will assess the impact of imputation*. We will repeat the comparison between intervention and control groups using only cases with postpartum follow-up. This will involve no imputation.

2.2.3.2 Complete postpartum follow-up (for all components of primary outcome):*This will assess the impact of our assumptions about missing components of the primary outcome*. Pregnancies for whom we have complete outcome data (i.e., all questions were answered for the primary outcome). This will involve no imputation. However, if this is significant, then we will consider repeating 2.2.3.1 with the imputation based only for women with 42 day postpartum follow-up.

2.2.3.3 Women whose EDD+3 weeks falls within the trial timeline: *This will assess the impact of the intervention independent of gestational age at birth*. This includes women for whom the primary outcome was imputed.

2.2.3.4 Women whose EDD+3 weeks plus 42 day follow up period falls within the trial timeline: *This will assess the impact of incomplete postpartum follow-up that was <42 days postpartum*. This will involve all women for whom the primary outcome was imputed.

2.2.3.5 Without adjustment:*This will assess the impact of adjustment.* Sensitivity analyses without adjustment at either the cluster or individual levels will be conducted. This will involve all women for whom the primary outcome was imputed.

2.2.3.6 Inclusion of women enrolled postpartum *This will assess the impact of postpartum enrollment of women.* Such women in the intervention arm may have been exposed to community engagement, however will not have CLIP antenatal visits guided by POM application.

Note to file: This change was made when it was recognised that women were also enrolled postpartum in Mozambique because of six-monthly surveillance cycles and cultural barriers (such as late disclosure of pregnancy).

2.2.3.7 Evaluating 'on treatment' effect: An evaluation of treatment effect will be undertaken comparing women with one or more CLIP visits in intervention arm to women in the control arm. Assessment of the effect of the penetration of the intervention in terms of intensity of community engagement and of the CLIP visits will take place in the process evaluation within the intervention clusters. We will be unable to look at the third aspect of the intervention (i.e., effect of compliance with treatment recommendations) given small numbers.

2.3. Application of the intervention

Application of the intervention and challenges

This analysis is justifiable because of challenges with application of the intervention. The following criteria may be used to define differing degrees of compliance:

Bone JN, *et al. BMJ Global Health* 2021; 6:e004123. doi: 10.1136/bmjgh-2020-004123

A. Community Engagement – There is no clear acceptable value for the measures of community engagement. As such, we will use the following measures to rate the degree of community engagement, according to 'higher' community engagement (i.e., median or higher event rate or value) or 'lower' community engagement (i.e., below median event rate or value) in intervention clusters:
   a. N (%) meetings held
   b. N (%) meetings at which each key individual topic was covered
   c. N attendees (i.e., large/small meetings)
   d. N meetings that included male decision-makers
   e. N meetings that included community leaders (male or female)
B. CLIP Visits and POM-guided management

Analyses will be done according to:

- N (%) of women who received a CLIP POM visit(s)
- N (%) women (of all those in the clusters who received CLIP POM visits at least once every 4 weeks and once postpartum, as specified in the study protocol
- N (%) women who accepted (as defined in Section 2.2.1) the POM-guided transport/treatment recommendation compared with those who were not, among women who were given a recommendation

Referral acceptance was determined based on confirmation at a follow-up visit that the woman received treatment and/or went to facility. The concordance between these initial and follow-up compliance responses will be used to describe compliance for women for whom there was no follow-up visit. Decision rule for POM acceptance of referral is shown in Table 5, data fields used in the analysis of POM acceptance of referral are shown in table 6.

Withdrawal from surveillance is described in Appendix 4.3.

2.4Secondary Analyses

2.4.1 Secondary outcomes

The secondary outcomes have been defined to measure the impact of the CLIP intervention on the delays around triage and transport.

(1) Birth preparedness and complication readiness, will assess the success of community engagement, and be measured by any *three* of the following, measured antenatally as close to delivery as available*:

(1) arranged for transport;

(2) obtained prior permission for transport should emergency arise;

(3) saved money for obstetric care;

(4) identified skilled birth attendant;

(5) identified facility for delivery.

**Any measurements taken postpartum will be disregarded*.

Bone JN, *et al. BMJ Global Health* 2021; 6:e004123. doi: 10.1136/bmjgh-2020-004123

(2) Proportion of women presenting for care at a CEmOC facility and proportion of facility births: (This number should increase if triage (using the miniPIERS risk stratification and POM) is effective, and if there is compliance with transport to CEmOC facility.)

Methods applied to the individual components of the primary outcome will be used for the individual components of the Secondary outcome. In addition, the proportion of women achieving birth preparedness and complication readiness will be compared between intervention and control clusters, as will be the proportion of women presenting for care at anCEmOC facility, and the proportion of facility births.

Table 8 shows the data fields used in the analysis of secondary outcomes.

2.4.2 Other Outcomes

- Individual components of the primary outcome, including N seizures (overall and either pre-CLIP visit or post-CLIP visit , pre- or post- MgSO4 administration where applicable), maternal death or morbidity timing relative to administration of antihypertensive therapy for severe hypertension
- Adverse events (see 2.6.1 below)
- Knowledge of pre-eclampsia/eclampsia
- Total number of: antenatal care sought, N blood pressure measurements performed by health professionals, antihypertensive therapy received
- Gestational age at delivery
- Functional disability, which is defined as the inability to perform usual daily duties at specific points in time during the postpartum period. These are defined to reflect what would be acceptable and expected culturally in each country. The normative ranges will be defined by data from the control clusters (We hypothesise that a threshold of 90th centile would be reflect return to functional ability in each of the sites, however this would be determined from the data)
- Other perinatal morbidities: neonatal fever, congenital anomaly and birth injury.

2.4.3 Other Planned secondary analysis

- Cost-effectiveness of the CLIP intervention (See Appendix 4.5 for details)
- Geo-temporal analysis (MOMI) (See Appendix 4.4 for details)

We will analyse the following subgroups by country and within each of the three countries, by cluster:

- Country
    - **Cultural beliefs and practices** (alternative medicine, religious beliefs, beliefs about the medical system)
- Clusters
    - **Size** (large vs. small) - The size of the clusters would be obtained from national census data
    - **Geographical/seasonal challenges** (e.g. rivers)
- Women

Bone JN, *et al. BMJ Global Health* 2021; 6:e004123. doi: 10.1136/bmjgh-2020-004123

- **Parity** (nulliparous vs. parous) - This analysis would be ascertained by evaluating parity (defined as deliveries at ≥20 wk and/or ≥500g)
- **Literacy** (literate vs. illiterate)
- **Distance that women live from the PHC** (close vs. far) - PHC Distance to the PHC may have to be ascertained using GIS mapping and analysis. This would limit this analysis to the time period of Definitive Trial and would be possible in two countries: Pakistan and Mozambique
- **Poverty index**
- **Gestational age at delivery**

- Community
    - **Community engagement**
        - **High vs. low** (defined as N meetings, N attendees/meeting, N topics covered)
    - **Community health workers**
        - Baseline level of education (defined as the entry requirement for the relevant training programme)
        - Intensity of initial training
        - Attendance at updates
    - **CLIP visits**
        - **Where the visits were conducted** (PHC vs. in the home)
        - **Intensity of CLIP visits**, measure of 'compliance' with the protocol, defined as women who had prescribed N antenatal visits based on gestational age at enrollment and delivery (vs. those who did not), and women who had prescribed N postnatal visits (vs. those who did not) - OR defined by quartiles of N visits/N weeks between enrollment and delivery.
        - **Clinical compliance** with CLIP recommendations for referral
            - Women who went to facility vs. those who did not
            - Women who were referred back to the community vs. those who were not
        - **Transport** (by ambulance, private car/taxi, other transport, walking)
- Health care system
    - **Density of community health care provider per population** – defined as the N population covered by each cHCP accounting for the new cHCP hired for CLIP
    - **Quality of care at PHC** - N health care providers at PHC-level
    - **Quality of care at referral facilities** - N attendees (total) at CPD events at referral facilities (total), N (%)

- Time (later vs. earlier)
    - **Definitive vs. pilot trials** (which would exclude Mozambique)
    - **Quartiles of study period**
    - **Temporal trends** (observed/expected)

2.5 Interim Analysis

An interim analysis is planned for each of the three sites once complete data (until 42 days postpartum) have been received for women making up half of the planned sample size for that country. In the event that the site is unable to reach planned sample size, due to logistical and pragmatic challenges, then the interim analysis will be conducted once complete pregnancies (until 42 days postpartum) are expected for women making up half of the projected sample size for that country. Projection of sample size for each country will be based on the observed recruitment rates in the Definitive trial as follows: 12 months of recruitment in Pakistan, 15 months of recruitment in India, and 11 months of recruitment in Mozambique. The average monthly recruitment will be calculated and applied to the remaining months of the trial, to obtain the final projected sample size. The interim analysis is reviewed by the Data Safety and Monitoring Board (DSMB).The stopping rule for both benefit and harm will require an observed difference between groups associated with an alpha <0.001. Reporting and handling of adverse events will be in accordance with Good Clinical Practice (GCP) guidelines.

2.6 Safety Data

Adverse events (AE) and Serious adverse events (SAE) will be compared between intervention and control clusters. Analysis will be for the composite (one/more of the events), as well as for each of the events.

2.6.1 Definition of Adverse Events (AEs)

- Following methyldopa administration in the community, a sBP<110mmHg on arrival at facility (maternal hypotension)
    - All cases of hypotension (reported in facility from community treatment in an intervention cluster) will be pulled and cross referenced with methyldopa administration in the same women to confirm if this AE was related to the treatment
- Following MgSO4 administration in the community, either respiratory depression, coma or death during transport, as diagnosed upon arrival at facility
- Transport-related injury (life or limb) or death during transport
- Injection site haematoma or infection (following either community or facility administration of i.m. MgSO4)
- ≥ 20% of women referred to facility being sent back to their communities without follow-up (monitoring community engagement and the CLIP POM)
    - This will be measured as follows:

Bone JN, *et al. BMJ Global Health* 2021; 6:e004123. doi: 10.1136/bmjgh-2020-004123

- Include only POM visits with referral that occurred >14 days prior
- This may be antepartum or postpartum visit
- Calculate percentage of POM visits with referral that did not result in any subsequent POM visit

2.6.2 Definition of Serious Adverse Events (SAEs)

Adverse events that meet all of the following criteria:

(i) Serious

(ii) Unexpected (in nature, severity, or frequency), and

(iii) Thought to be related to the study intervention

2.7 Extraction of per-pregnancy data

Trial participants may provide data through multiple surveys. To analyze data on a per-pregnancy level, the following rules will be used to extract the data per pregnancy for analysis:

- For variables that record the presence ("Yes") or absence ("No") of a condition, the analyzed value will be "Yes" if this was answered in any of the forms, "No" if this and/or "unknown" was the only answer in any of the forms, and lastly "unknown" if this was the only answer in any of the forms
    - e.g. Dialysis (Yes/No/Unknown)
- For variables that record a choice out of a list of equally weighted options, and there can only be one answer per woman, the analysed value will be the last value entered
    - e.g. Delivery location (Home/Facility/On route/Other)
- For variables that record a choice out of a list of equally weighted options, and there can multiple answers per woman, each survey response will be analysed
    - e.g. Type of facility visited during the most recent admission
- For variables that record a numeric value, and there can only be one answer per woman, the analysed value will be the last value entered
    - e.g. Age
- For variables that record a frequency value, that is recorded at each survey, the analysed value will be the summation of all values entered
    - e.g. Number of visits to hospital

2.8 Individual patient data meta-analysis (IPD)

At the completion of all three trials (Mozambique, Pakistan and India), an individual patient data (IPD) meta-analysis will be conducted (at UBC). For the IPD meta-analysis, we will analyse data for women recruited at ≥20 weeks, to standardise data to the latest public declaration of pregnancy (i.e., 20 weeks in Pakistan). If baseline outcome data are available and consistent across all three countries, these data will be used for adjustment in analysis. For power calculation, see Appendix section 4.2.

3.0 Additional Methodological Details

3.1 Statistical Tests

Outcome rates will be compared using measures of effect size (difference in event rates, relative risks, etc) with 95% confidence intervals. Where needed, statistical tests will be two-sided, with significance levels of 0.05 for primary outcome, and 0.001 for secondary and other outcomes, unless stated otherwise.

The population standardized risk difference will be presented as an alternate summary of the benefit of the intervention rather than as a primary analytic endpoint.

3.2 Software to be Used

SAS / R.

Bone JN, *et al. BMJ Global Health* 2021; 6:e004123. doi: 10.1136/bmjgh-2020-004123

4. Appendix

4.1 CLIP country trials Sample Size Calculations

Country	Mozambique	Pakistan	India
Unit of randomization	AP	Union Council	PHC
Population per unit of randomization	25,000	32,000	27,000
Annual birth rate (/1000/yr)	40	14	22
Births/2yr	2000	900	1200
MMR (/100,000 live births)	388	267	150
Intra-cluster co-efficient	0.002‡	0.002*	0.001**
Incidence of maternal & perinatal/neonatal M&M<br>control clusters<br>intervention clusters	 14.00%<br>11.10%	 9.60%<br>7.70%	 5.40%<br>4.30%
Number of clusters (total)	12	20	12
Number of births in Definitive CLIP Trial<br>(2 years)	24,000	18,000	14,400
Additional births from Pilot CLIP Trial<br>(4 clusters/1 year)	0	1800	2400
Total number of births<br>(Pilot & Definitive Trials)	24,000	19,800	16,800
Expected referrals at month 6 of Pilot Trial assuming 5% incidence of HDP	100 [81, 119]	45 [32, 58]	60 [45, 75]
Number of women to be referred at month 6 of Pilot Trial to meet 50% use target	50	23	30
Number of health facilities (total)<br>PHC/RHC<br>EmOC	56<br>10 BEmoc<br>1 CEmoc	 42 	 22<br>22

**Number of cHCPs (total)**	135 APEs (71 prior to study start)	400 LHWs	260<br>(215 ASHAs, 45 ANMs)

ANM: assistant nurse midwife; **AM: Administrative Posts; ASHA**: accredited social health activist; **CHEW**: community health extension worker; **cHCPs**: community health care providers; **CHW** community health worker; **EmOC**, emergency obstetric care; **LGA**: Local Government Area; **LHW**: Lady Health Worker; **M&M**: morbidity and mortality; **MMR**: maternal mortality ratio; **PHC**: Primary Health Centre; **RHC**, rural health centre.

* calculated from Sindhi miniPIERS facility data; ‡ assumes same ICC as Sindh (urban population in Maputo); ** assumes half the ICC of Sindh. In these calculations, we have used risks at the lower end of the published ranges for that risk and milieu.

Assumptions:

- 10% loss of individual women to follow-up
- No loss of clusters to follow-up
- Alpha of 0.05, power ≥0.80
- Anticipated effect size of a 20% reduction in all cause maternal and perinatal/neonatal morbidity and mortality
- Also, we have assumed a ratio of maternal morbidity : mortality of 5:1 (may be as high as 10:1), and a ratio of perinatal/neonatal : maternal events of 5:1 (may be as high as 10:1 – permits overlap in outcomes)
- All sample sizes will be supplemented by the data collected in each single country Pilot CLIP Trial.

4.2 CLIP IPD Meta-analysis Power Calculations

The combined Definitive CLIP Trials (Mozambique, Pakistan and India) cohort of 60,600 deliveries in 44 clusters over 2 years, averaging 1377 deliveries per cluster per year, will provide the following power for the IPD meta-analysis:

- CLIP Definitive Trials Primary Outcome (Maternal & fetal/neonatal mortality & morbidity): assuming a 20% effect size (incidence reduced from 10.2% to 8.2%), we would have 80% power with an ICC of 0.006.
- Maternal mortality & morbidity (primary outcome for the IPD meta-analysis): assuming a 20% effect size (incidence reduced from 1.7% to 1.4%), we would have 60% power with an ICC of 0.001. To achieve 80% power will require an ICC of <0.001 which is unlikely.
- Maternal mortality: assuming a 30% effect size (incidence reduced from 1.7% to 1.2%), we would have 80% power with an ICC of 0.002. We hope to reduce the adverse maternal event rate by ≥30%, primarily through community mobilisation and antenatal care by cHCPs.

4.3 Withdrawal

- **Mozambique:** a woman who has received a Form D (pregnancy registration), who rejects further trial surveillance (Form E). In intervention clusters, there is then consent for POM and the package of care in response to a CLIP trigger, should one develop.
- **India:** Withdrawal is defined as a woman with a MN01 who declines having MN02. In intervention clusters, there is then consent for POM and the package of care in response to a CLIP trigger, should one develop.
- **Pakistan**: Withdrawal is defined as a woman with pregnancy registration who declines to have trial surveillance (antepartum/postpartum). In intervention clusters, there is then consent for POM, and then should a CLIP trigger occur, consent for the package of care

4.4 The Geo-Temporal analysis

The Geo-Temporal analysis will serve to; 1) elucidate the spatial variation in community level risk for adverse maternal outcomes, 2) Identify the factors that promote maternal resilience in the different communities under study, and 3) illustrate changing rates of adverse outcomes through the timeline of the CLIP trials. The population level estimates of variables of interest will be calculated using data from the surveillance cycles, while community rates of adverse outcomes will be calculated by combining the maternal deaths and severe morbidities recorded through facility surveillance. Geographically Weighted Regression (GWR) will be used showcase the spatial variation in associations between the choice variables, and rates of adverse maternal outcomes. Communities with less than expected rates of adverse outcomes, based on their community characteristics, will be further assessed using GWR against the predetermined community resilience variables to identify the factors that are associated with maternal resilience. Key place specific factors promoting maternal resilience will we weighted and combined into an index measuring maternal resilience. Summaries of predetermined indicators of both primary and secondary outcomes of the trial will be calculated and mapped using the ArcGIS software on a quarterly basis for the entire timeline of the trial data. This will enable the creation of geo-temporal visualizations and data indicating the changing rates of outcomes throughout the timeline of the trial.

4.5 Health Economics – Plan of analysis

Parameter estimates for costs and effectiveness will be derived from the CLIP surveillance questionnaire. The unit costs will be multiplied by identified health resource utilization to calculate the total cost per pregnancy, including both pregnant woman and newborns. The total cost will be calculated as the sum of the health resource utilization cost, cost of implementing the CLIP package of care, cost of routine pregnancy care and societal costs. The annual equivalent costs in local currency of selected CLIP countries will be converted to US dollar. The pregnancy outcomes (i.e. health of mother and baby) will be modelled as the effectiveness of the CLIP interventions. This will include no-adverse outcomes (healthy mother and newborn at the time of delivery) and adverse outcomes (death and/or disability of mother and baby) observed in the intervention and control groups. Using the data and parameter estimates specific to each CLIP country, the incremental cost-effectiveness ratios (ICER)s will be calculated first from a healthcare system perspective and then from a societal perspective. The country-specific ICER will be calculated as incremental cost per adverse pregnancy outcome. Comparison will be made between the country-specific ICER and the per capita value for the gross national income for the year 2015.

Given the uncertainties involved in CEA, we will use probabilistic sensitivity analysis to produce cost-effectiveness plots. The confidence region surrounding the cost-effectiveness ratio will be estimated using appropriate statistical methods, including bootstrap and Monte Carlo simulations. Life tables based on data from the World Health Organization's Southeast Asia and African regions or the West level-26 model will also be used in a sensitivity analysis. Country-specific health system budget impact analysis will be conducted to facilitate policy decisions for resource allocation.

Our primary analysis for this study will be model based, guided by previous work in high-income countries as no LMIC modelling in pre-eclampsia has been done. We will use parameter estimates for costs and effectiveness coming from the CLIP Trial (see Figure1). The unit costs will be multiplied by identified health resource utilization to calculate the total cost per pregnancy, including both pregnant woman and newborns. The total cost will be calculated as the sum of the health resource utilization cost, cost of implementing the CLIP package of care, cost of routine pregnancy care and societal costs. The annual equivalent costs in local currency of selected CLIP countries (PKR—Pakistani Rupee; INR—Indian Rupee, NGN—Nigerian Naira and MZN—Mozambican Metical) will be converted in US dollar exchange rate as of 2015. The pregnancy outcomes (i.e. health of mother and baby) will be modelled as the effectiveness of the CLIP interventions. This will include no-adverse outcomes (healthy mum and newborn at the time of delivery) and adverse outcomes (death and/or disability of mother and baby) observed in the intervention and control groups. Using the data and parameter estimates specific to each CLIP country, the ICERs will be calculated first from a healthcare system perspective and then from a societal perspective. The country-specific ICER will be calculated as Incremental cost per adverse pregnancy outcome. ICERs for the system perspective as the reference case will be of interest to country-specific health policy makers for resource allocation decisions, when switching from routine pregnancy care to CLIP plus routine pregnancy care, should CLIP be found effective. Critically, however, the ICER from a societal perspective will facilitate discourse on the full opportunity cost in the context of the selected CLIP country. In accord with the recommendation of the Commission for Macroeconomics and Health, we will compare the country-specific ICER with the per capita value for the gross national income of each of the four selected CLIP countries for the year 2015.

Given the uncertainties involved in CEA, we will use probabilistic sensitivity analysis to produce cost-effectiveness plots. The confidence region surrounding the cost-effectiveness ratio will be estimated

Bone JN, *et al. BMJ Global Health* 2021; 6:e004123. doi: 10.1136/bmjgh-2020-004123

using appropriate statistical methods, including bootstrap and Monte Carlo simulations. Life tables based on data from the World Health Organization's Southeast Asia and African regions or the West level-26 model will also be used in a sensitivity analysis. Children in LMICs bear a disproportionately large share of the total disease burden, because of the cause structure of the disease burden by age could influence overall distribution of DALYs. As reported on previous cost-effectiveness studies in LMIC, no-age-weighting in the reference case was used on sensitivity analysis. Country-specific health system budget impact analysis will be conducted to facilitate policy decisions for resource allocation, when switching from routine pregnancy care to CLIP plus routine care, should the intervention be found effective.

Figure 1 (next page) Decision analytic tree model for economic evaluation of CLIP. HDP, hypertensive disorder of pregnancy

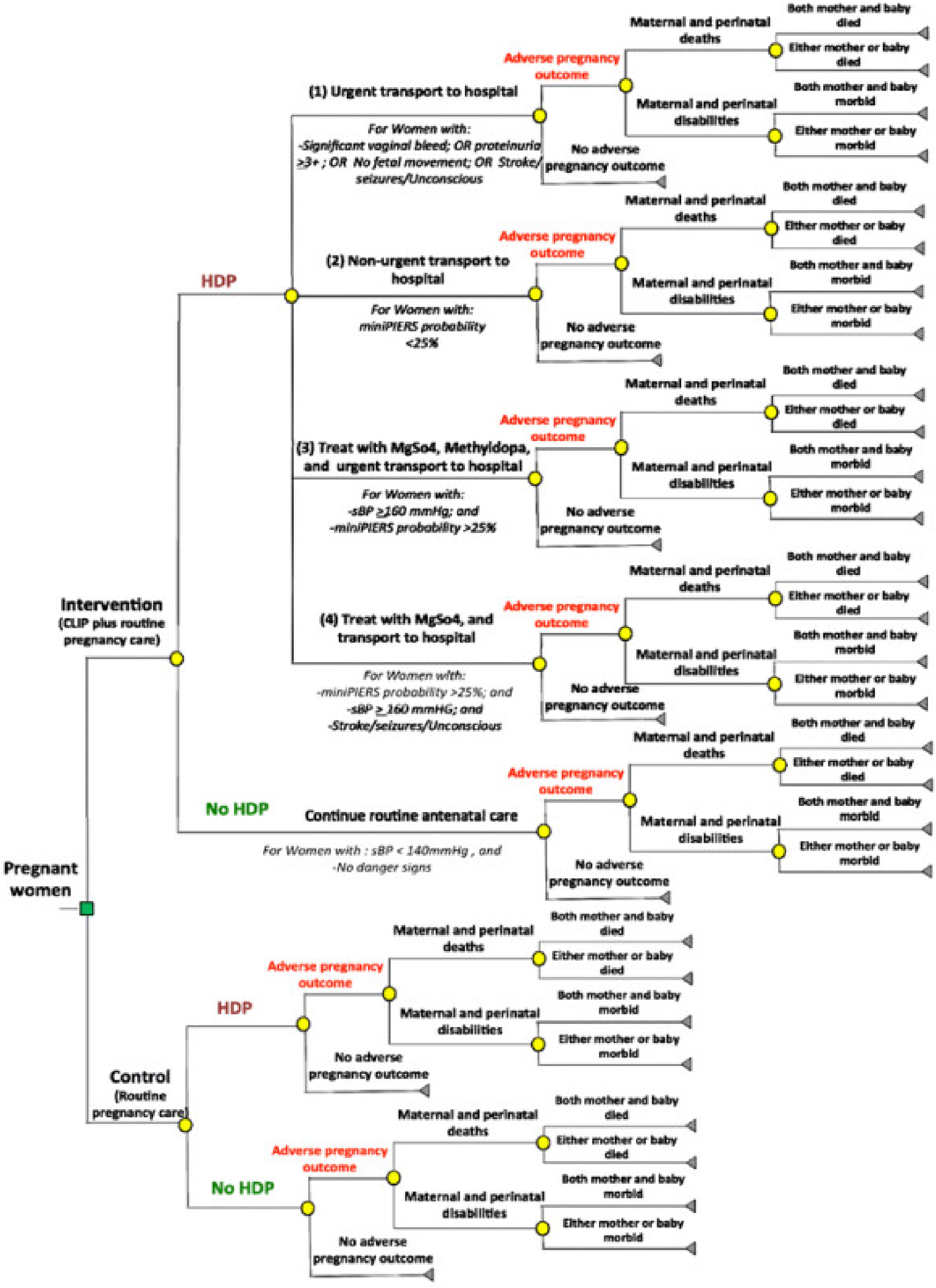

HDP = Hypertensive Disorder of Pregnancy

BMJ Publishing Group Limited (BMJ) disclaims all liability and responsibility arising from any reliance placed on this supplemental material which has been supplied by the author(s)

Tables in CLIP Statistical Analysis Plan

Table 1 Design of the Pilot Trial 1

Table 2 Design of the Definitive Trial.. 2

Table 3 Data fields used in the analysis of baseline and demographic variables 3

Table 4 Baseline outcome variables collected, by country 4

Table 6 Data fields used in the analysis of POM acceptance of referral 5

Table 7 Data fields used in the analysis of primary outcome.. 6

Table 8 Data fields used in the analysis of secondary outcomes 7

Table 9 Data fields used in the analysis of other outcomes.. 7

Table 10 Data fields used in the analysis of adverse events 9

Table1 Design of the CLIP Pilot Trials

	**Pakistan**<br>(Hyderabad and Matiari districts in Sindh Province)	**India**<br>(Belgaum and Bagalkot districts in Karnataka State)
**Timeline**	Feb 2014 – Dec 2014	Feb 2014 – Oct 2014
**Number of control clusters**	2	2
**Number of intervention clusters**	2	2
**Data collection**	Ongoing pregnancy registration;<br>Regular household surveys on quarterly basis;<br>Facility surveillance in public tertiary level hospitals only	Using MNH Registry with three time points of data collection:<br>Pregnancy identification;<br>Within 7 days of delivery;<br>42 days postpartum

Table 2 Design of the CLIP Definitive Trials

	**Mozambique**<br><br>(Maputo and Gaza Province)	**Pakistan**<br>(Hyderabad and Matiari districts in Sindh Province)	**India**<br>(Belgaum and Bagalkot districts in Karnataka State)
**Timeline**	Feb 2015 -	Mar 2015 -	Nov 2014 -
**Number of control clusters**	6	10	6
**Number of intervention clusters**	6	10	6
**Data collection**	Ongoing pregnancy registration;<br>Regular household survey every six months with ongoing facility data collection;<br>Maternal and Perinatal verbal autopsies for all deaths	Ongoing pregnancy registration;<br>Regular household surveys on quarterly basis;<br>Facility surveillance in public tertiary level hospitals only;<br>Maternal and Perinatal verbal autopsies for all deaths	Using MNH Registry with three time points of data collection:<br>Pregnancy identification;<br>Within 7 days of delivery;<br>42 days postpartum

Table 3 Data fields used in the analysis of baseline and demographic variables

CRF	Section	Field
Pregnancy registration	Demographic information	• Age<br>• Years of school<br>• Partner in house (Mozambique only)
Pregnancy registration	Obstetric history	• Parity<br>• High blood pressure<br>• Seizures
Pregnancy registration	Gestational age assessment	• EDD / LMP
Pregnancy registration	Medical history	• High blood pressure<br>• Seizures
Regular surveillance	Cost of newborn interventions / admissions	• Time to facility
Baseline survey	Household information	• Poverty index

Bone JN, *et al. BMJ Global Health* 2021; 6:e004123. doi: 10.1136/bmjgh-2020-004123

Table 4 Baseline outcome variables collected, by country

	**Pakistan**	**Mozambique**	**India**
N MWRA (married women of reproductive age)	x	x	x
Age of MWRA	x	x	x
Number of MWRA with one or more delivery/ abortion reported in last 12 months (n(%))	x	x	x
Number of pregnancies resulting in a live birth (n(%))	x	x	x
Number of pregnancies resulting in a still birth (n(%))	x	x	x
Number of pregnancies resulting in a miscarriage (n(%))	x	x	x
Pregnancies complicated by high blood pressure (n(%))	x	x	x
Pregnancies complicated by eclampsia(n(%))	x		x
Pregnancies complicated by seizure(n(%))		x	
Pregnancies complicated by severe headache (n(%))	x		
MRWA deaths reported n(%)	x	x	x
Maternal deaths		x	x
Age of woman at death (mean +/- sd)	x	x	x
**Children deaths within 1 week (early neonatal death)**	**x**	**x**	**x**
**Children deaths between 1 week and 1 month (late neonatal death)**	**x**	**x**	**x**
Maternal morbidity			x
Blood transfusion			x
Hysterectomy			x
Antepartum haemorrhage			x
Coma			x
Failure to form clots			x
Fever & symptom			x
Headache and stiff neck			x
Cough and shortness of breath			x
Abd pain or uterine tenderness			x
Flank pain			x
Foul smelling vag discharge			x
Received Anti-shock garment			x
Received Bimanual uterine compression			x
Received Brace sutures			x
Received CPR			x
Received Dialysis			x
Received Internal iliac artery ligation/devascularisation procedure			x
Received Mechanical ventilation			x
Seizure (fits)			x
Stroke			x

Neonatal Morbidity			x
Breathing problems			x
Feeding problems			x
Lethargy			x
Coma (neonatal)			x
Seizure (neonatal)			x
Umbilical stump infection			x
Skin infection			x
Jaundice			x
Diarrhea/vomiting			x
Bleeding			x

Table 5 Decision rule for POM acceptance of referral

**Follow-up response (Did you go to hospital?):**	**Yes**			**No**		**Missing**			
**Response given immediately following recommendation:**	**Accept**	**Refuse**	**Missing**	**Accept**	**Refuse**	**Missing**	**Accept**	**Refuse**	**Missing**
Acceptance	√	√	√	X	X	X	√	X	Missing

Table 6 Data fields used in the analysis of POM acceptance of referral

**CRF**	**Section**	**Field**
PIERS on the Move	Visit	• Transport
PIERS on the Move	Follow-up	• Went to hospital after last home visit

Table 7 Data fields used in the analysis of primary outcome

CRF	Section	Field
Regular surveillance	Demographic information	• Deceased
Regular surveillance	Maternal conditions and interventions	• Cardiopulmonary resuscitation (CPR)<br>• Mechanical ventilation<br>• Dialysis<br>• Blood transfusion<br>• Disseminated intravascular coagulation (DIC)<br>• Antepartum haemorrhage<br>• Anti-shock garment<br>• Hysterectomy<br>• Stroke<br>• Seizure<br>• Coma<br>• Obstetric sepsis
Regular surveillance	Perinatal outcomes	• Birth outcome<br>• Death within one week of birth<br>• Death between one week and one month of age
Regular surveillance	Perinatal outcomes	• Feeding difficulty<br>• Breath difficulty<br>• Seizure<br>• Lethargy<br>• Coma<br>• Hypothermia<br>• Umbilical cord infection<br>• Skin infection<br>• Bleeding<br>• Jaundice<br>• Central nervous system related morbidity

Table 8 Data fields used in the analysis of secondary outcomes

CRF	Section	Field
Regular surveillance	Delivery information	• Location of delivery
Regular surveillance	Birth preparedness	• Where to deliver<br>• Skilled birth attendant<br>• Transportation plan<br>• Funds saved for emergencies<br>• Permission to go seek care
Regular surveillance	Care-seeking behaviour	• Care sought from health care provider<br>• Visits to primary health centres<br>• Visits to higher level facilities

Table 9 Data fields used in the analysis of other outcomes

CRF	Section	Field
Regular surveillance	Delivery information	• Location of delivery<br>• Blood pressure measured<br>• Antihypertensive medications
Regular surveillance	Pre-eclampsia knowledge	• Abnormal bleeding after delivery<br>• High blood pressure during pregnancy<br>• High blood pressure can cause death of pregnant women<br>• Seizures during pregnancy<br>• Headache<br>• Visual disturbance<br>• Chest pain<br>• Shortness of breath<br>• Nausea and vomiting<br>• Abdominal pain<br>• Vaginal bleeding

CRF	Section	Field
		• Unconsciousness<br>• Stroke<br>• Seizures<br>• Unusual swelling
Regular surveillance	Medications and adverse effects	• Received MgSO4
PIERS on the Move	Visit	• MgSO4
PIERS on the Move	Follow-up	• Followup_MgSO4<br>• Haematoma<br>• Infection
Regular surveillance	Functional disability	• Take care of newborn<br>• Wash babies' clothes<br>• Prepare meals<br>• Clean the house<br>• Get water<br>• Work in the fields<br>• Go to the market<br>• Return to work/paid employment

Bone JN, *et al. BMJ Global Health* 2021; 6:e004123. doi: 10.1136/bmjgh-2020-004123

Table 10 Data fields used in the analysis of adverse events

CRF	Section	Field
PIERS on the Move	Visit	• Methyldopa<br>• MgSO4<br>• Visit date
PIERS on the Move	Follow-up	• Physical injury during transport<br>• Haematoma at injection site<br>• Infection at injection site
Facility Surveillance	Visit information	• Reason for seeking care<br>• Status on arrival
Facility Surveillance	Clinical characteristics on arrival	• Blood pressure
Facility Surveillance	MgSO4-related adverse effects	• Haematoma at injection site<br>• Infection at injection site

Bone JN, *et al. BMJ Global Health* 2021; 6:e004123. doi: 10.1136/bmjgh-2020-004123
