# Supplementary material for: Economic and cost-effectiveness analysis of the Community-Level Interventions for Pre-eclampsia (CLIP) trials in India, Pakistan and Mozambique
Source: BMJ Glob Health. 2021 May 24;6(5):e004123. doi: 10.1136/bmjgh-2020-004123 (PMC8149358; doi:10.1136/bmjgh-2020-004123)

Supplementary Table 1: CLIP Trials working group

**CLIP Trials Working Group**
Esperança Sevene, Eusébio Macete, Khátia Munguambe, Charfudin Sacoor, Anifa Vala, Helena Boene, Felizarda Amose, Rosa Pires, Zefanias Nhamirre, Marta Macamo, Rogério Chiaú, Analisa Matavele, Faustino Vilanculo, Ariel Nhancolo, Silvestre Cutana, Ernesto Mandlate, Salésio Macuacua, Cassimo Bique, Sibone Mocumbi, Emília Gonçálves, Sónia Maculuve, Ana Ilda Biz, Dulce Mulungo, Orvalho Augusto, Paulo Filimone, Vivalde Nobela, Corsino Tchavana, Cláudio Nkumbula
Rahat Qureshi, Zulfiqar A Bhutta, Zahra Hoodbhoy, Farrukh Raza, Sana Sheikh, Javed Memon, Imran Ahmed, Amjad Hussain
Mrutunjaya B Bellad, Umesh S Charantimath, Shivaprasad S Goudar, Geetanjali M Katageri, Avinash J Kavi, Amit P Revankar, Ashalata A Mallapur, Umesh Y Ramdurg, Shashidhar G Bannale, Vaibhav B Dhamanekar, Geetanjali I Mungarwadi, Narayan V Honnungar, Bhalachandra S Kodkany, Anjali M Joshi, Uday S Kudachi, Sphoorthi S Mastiholi, Chandrappa C Karadiguddi, Gudadayya S Kengapur, Namdev A Kamble, Keval S Chougala
Jeffrey Bone, Dustin T Dunsmuir, Sharla K Drebit, Chirag Kariya, Mai-Lei Woo Kinshella, Tang Lee, Jing Li, Mansun Lui, Beth A Payne, Diane Sawchuck, Sumedha Sharma, Domena K Tu, Marianne Vidler, Ugochi V·Ukah, Laura A Magee, Peter von Dadelszen
**CLIP Trial Steering Committee**
J Mark Ansermino, Ana Pilar Betrán, Richard Derman, Shafik Dharamsi, France Donnay, Sharla Drebit, Guy Dumont, Susheela M Engelbrecht, Veronique Fillipi, Tabassum Firoz, William Grobman, Marian Knight, Ana Langer, Simon Lewin, Gwyneth Lewis, Craig Mitton, Nadine Schuurman, Andrew Shennan, Joel Singer, Jim Thornton, Hubert Wong
**CLIP Trial Executive Committee**
Olalekan O Adetoro, Mrutunjaya M Bellad, Zulfiqar A Bhutta, Peter von Dadelszen, Shivaprasad S Goudar, Laura A Magee, Ashalata A Mallapur, Khátia Munguambe, Beth A Payne, Rahat Qureshi, Charfudin Sacoor, Esperança Sevene, Sumedha Sharma, John O Sotunsa, Marianne Vidler
**CLIP Data Safety ansd Monitoring Board (DSMB)**
Romano Nkumbwa Byaruhanga, Brian Darlow, Eileen Hutton, Mario Merialdi, Lehana Thabane

Supplementary Table 2: Details of costs/number units ordered. All unit costs are taken from annual trial spending reports.

<table>
<tr><th></th><th colspan="2">India</th><th colspan="2">Mozambique</th><th colspan="2">Pakistan</th></tr>
<tr><td></td><td>Quantity</td><td>Total cost (USD)</td><td>Quantity</td><td>Total cost (USD)</td><td>Quantity</td><td>Total cost (USD)</td></tr>
<tr><td>Supplies</td><td>-</td><td>-</td><td></td><td></td><td></td><td></td></tr>
<tr><td>Digital blood pressure device</td><td>220</td><td>4,884</td><td>200</td><td rowspan="2">8040</td><td>390</td><td>8658</td></tr>
<tr><td>Large size blood pressure cuff</td><td>94</td><td>1,053</td><td>200</td><td>197</td><td>2207</td></tr>
<tr><td>Mobile device (Karabonn - A2+)</td><td>105</td><td>7,600</td><td>-</td><td>-</td><td>-</td><td>-</td></tr>
<tr><td>Mobile device(Samsung GT7262)</td><td>145</td><td>12,543</td><td>-</td><td>-</td><td>-</td><td>-</td></tr>
<tr><td>Mobile device(Samsung J1 Ace)</td><td>16</td><td>1,573</td><td>-</td><td>-</td><td>-</td><td>-</td></tr>
<tr><td>Mobile device (Model Q-Noir A7 phone)</td><td>-</td><td>-</td><td>-</td><td>-</td><td>92</td><td>11555</td></tr>
<tr><td>Mobile device (Model Q-300 tablet 7.0”)</td><td>-</td><td>-</td><td>-</td><td>2965</td><td>566</td><td>80938</td></tr>
<tr><td>Mobile device (Huawei media pad youth)</td><td>-</td><td>-</td><td>-</td><td>11060</td><td>-</td><td>-</td></tr>
<tr><td>Mobile device (Android 3G+W)</td><td>-</td><td>-</td><td>-</td><td>3997</td><td>-</td><td>-</td></tr>
<tr><td>PX-13-B Kenek pulse oximeter device</td><td>-</td><td>-</td><td></td><td></td><td>377</td><td>7578</td></tr>
<tr><td>Urine specimen cups</td><td>100</td><td>133</td><td>-</td><td rowspan="8">19322</td><td>7,500</td><td>759</td></tr>
<tr><td>Urine dipsticks</td><td>160</td><td>1,187</td><td>-</td><td>10,500</td><td>1754</td></tr>
<tr><td>Alcohol swabs</td><td>60</td><td>230</td><td>-</td><td>1500</td><td>26</td></tr>
<tr><td>Syringes (10ml) and Needles (23 gram)</td><td>100</td><td>92</td><td>100</td><td>1400</td><td>105</td></tr>
<tr><td>Hand-gloves</td><td>5</td><td>140</td><td>600</td><td>800</td><td>23</td></tr>
<tr><td>Needle cutters</td><td>75</td><td>69</td><td>-</td><td>33</td><td>79</td></tr>
<tr><td>Disposal/sharp boxes</td><td>150</td><td>70</td><td>-</td><td>42</td><td>25</td></tr>
<tr><td>CLIP-card with polythene bag</td><td>50 packs</td><td>238</td><td>-</td><td>16 packs</td><td>427</td></tr>
<tr><td>Drugs</td><td></td><td></td><td></td><td></td><td></td><td></td></tr>
<tr><td>Magnesium sulphate (10ml)</td><td>2350</td><td>1,115</td><td>-</td><td>4101</td><td>1210</td><td>145</td></tr>
<tr><td>Methyldopa (250mg)</td><td>1,000</td><td>250</td><td>-</td><td>3062</td><td>1900</td><td>67</td></tr>
<tr><td>CHW workers</td><td></td><td></td><td></td><td></td><td></td><td></td></tr>
<tr><td>Incentives</td><td>-</td><td>39,043</td><td>-</td><td>3097</td><td></td><td>79398*</td></tr>
<tr><td>Training for CLIP implementation</td><td>-</td><td>12,755</td><td>-</td><td>53205</td><td></td><td></td></tr>
<tr><td>Community engagement</td><td>-</td><td>7,048</td><td>-</td><td>9201</td><td>-</td><td>71942</td></tr>
<tr><td>Out of pocket costs</td><td>-</td><td>-</td><td>-</td><td>-</td><td>-</td><td>-</td></tr>
<tr><td>Control (per pregnancy)</td><td>-</td><td>-</td><td>-</td><td>-</td><td>-</td><td>158**</td></tr>
<tr><td>Intervention (per pregnancy)</td><td>-</td><td>-</td><td>-</td><td>-</td><td>-</td><td>156**</td></tr>
</table>

*Cannot be disaggregated

** Estimated via a mixed effects linear model

Supplementary Table 3: Assumed probability distributions for probabilistic sensitivity analysis for each node of the decision tree.

Variable	Assumed distribution	Assumed distribution	Assumed distribution
**POM visits**	Dirichlet(p_1, p_2, p_3, p_4)	Dirichlet(p_1, p_2, p_3, p_4)	Dirichlet(p_1, p_2, p_3, p_4)
0			
1-3			
4-7			
≥8			
**Use of MgSO4/methyldopa**			
0	Beta(α, β)	Beta(α, β)	Beta(α, β)
1-3	Beta(α, β)	Beta(α, β)	Beta(α, β)
4-7	Beta(α, β)	Beta(α, β)	Beta(α, β)
≥8	Beta(α, β)	Beta(α, β)	Beta(α, β)
**Costs by POM visit**			
0	Gamma(α, β)	Gamma(α, β)	Gamma(α, β)
1-3	Gamma(α, β)	Gamma(α, β)	Gamma(α, β)
4-7	Gamma(α, β)	Gamma(α, β)	Gamma(α, β)
≥8	Gamma(α, β)	Gamma(α, β)	Gamma(α, β)
**Intervention outcomes**			
Maternal mortality	Beta(α, β)	Beta(α, β)	Beta(α, β)
Stillbirth	Beta(α, β)	Beta(α, β)	Beta(α, β)
Neonatal mortality	Beta(α, β)	Beta(α, β)	Beta(α, β)
**Control outcomes**			
Maternal mortality	Beta(α, β)	Beta(α, β)	Beta(α, β)
Stillbirth	Beta(α, β)	Beta(α, β)	Beta(α, β)
Neonatal mortality	Beta(α, β)	Beta(α, β)	Beta(α, β)

Supplementary Table 4: Probabilities of cost effectiveness based on country specific willingness to pay thresholds based on 3x the country GDP/capita for overall and various POM guided visit scenarios compared to control from probabilistic sensitivity analysis.

	**India (program) (WTP = $5188)**	**Mozambique (program) (WTP = $1287)**	**Pakistan (program) (WTP = $4105)**	**Pakistan (societal) (WTP = $4105)**
Overall	40.0%	35.0%	66.3%	66.9%
0 POM guided contacts	47.6%	61.8%	46.9%	47.1%
1-3 POM guided contacts	8.6%	0.0%	0.0%	5.5%
4-7 POM guided contacts	0.0%	1.8%	99.7%	99.8%
≥8 POM guided contacts	90.0%	96.3%	100%	100%

Supplementary Figure 1: Decision tree example (similar for other numbers of POM guided contacts/branches)

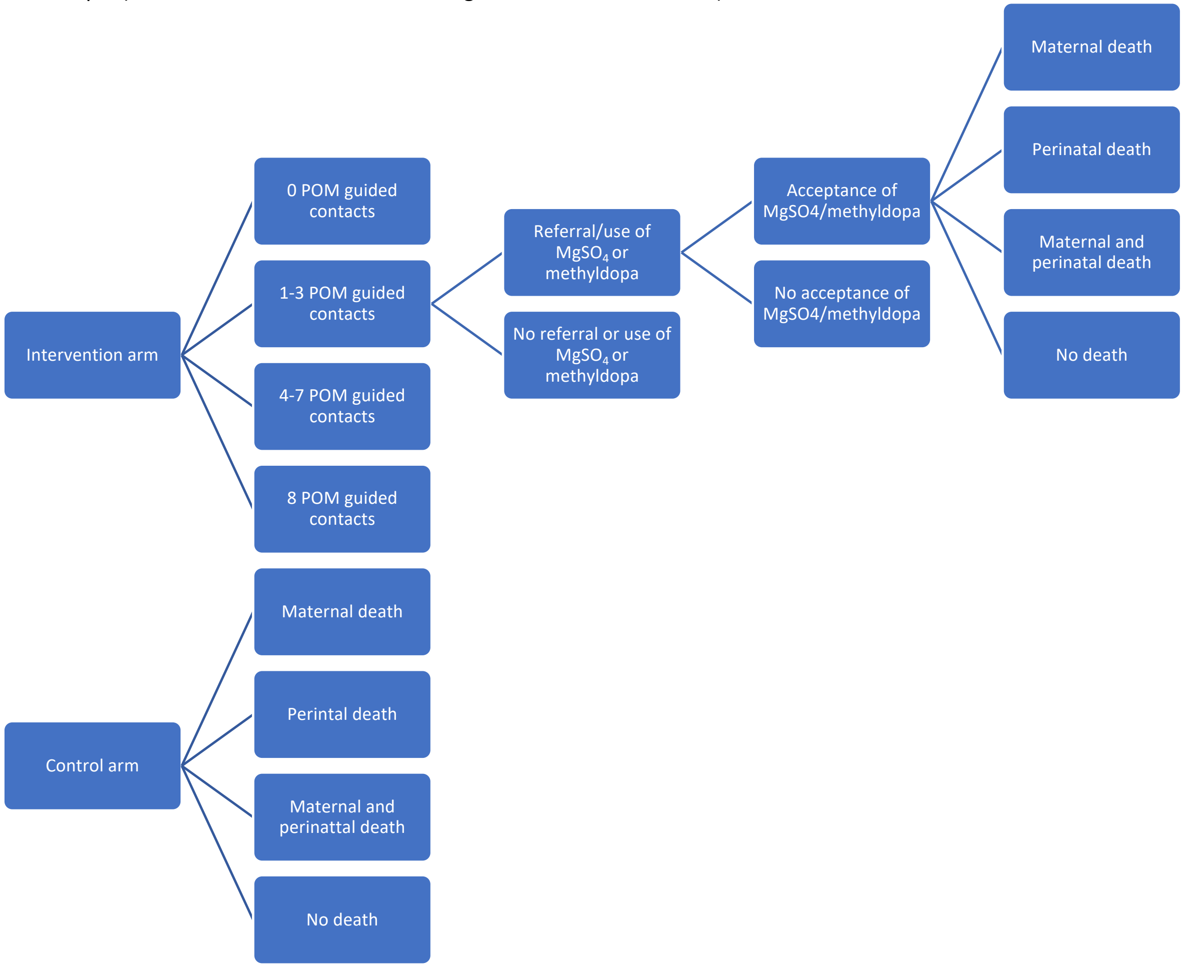

Supplement: Supplementary data [file bmjgh-2020-004123supp002.pdf]
